# Dioscin alleviates alcoholic liver fibrosis by attenuating hepatic stellate cell activation via the TLR4/MyD88/NF-κB signaling pathway

**DOI:** 10.1038/srep18038

**Published:** 2015-12-10

**Authors:** Min Liu, Youwei Xu, Xu Han, Lianhong Yin, Lina Xu, Yan Qi, Yanyan Zhao, Kexin Liu, Jinyong Peng

**Affiliations:** 1 College of Pharmacy, Dalian Medical University, No. 9 West Part of Lvshunnan Road, Dalian 116044, China

## Abstract

The present work aimed to investigate the activities and underlying mechanisms of dioscin against alcoholic liver fibrosis (ALF). *In vivo* liver fibrosis in mice was induced by an alcoholic liquid diet, and *in vitro* studies were performed on activated HSC-T6 and LX2 cells treated with lipopolysaccharide. Our results showed that dioscin significantly attenuated hepatic stellate cells (HSCs) activation, improved collagen accumulation, and attenuated inflammation through down-regulating the levels of myeloid differentiation factor 88 (MyD88), nuclear factor κB (NF-κB), interleukin (IL)-1, IL-6 and tumour necrosis factor-α by decreasing Toll-like receptor (TLR)4 expression both *in vivo* and *in vitro*. TLR4 overexpression was also decreased by dioscin, leading to the markedly down-regulated levels of MyD88, NF-κB, transforming growth factor-β1 (TGF-β1), α-smooth muscle actin (α-SMA) and type I collagen (COL1A1) in cultured HSCs. Suppression of cellular MyD88 by ST2825 or abrogation of NF-κB by pyrrolidine dithiocarbamate eliminated the inhibitory effects of dioscin on the levels of TGF-β1, α-SMA and COL1A1. In a word, dioscin exhibited potent effects against ALF via altering TLR4/MyD88/NF-κB signaling pathway, which provided novel insights into the mechanisms of this compound as an antifibrogenic candidate for the treatment of ALF in the future.

Alcoholic liver diseases (ALDs) including alcoholic fatty liver, alcoholic liver fibrosis (ALF) and alcoholic cirrhosis are among the most common and critical complications of heavy alcohol drinking^1^,^2^,^3^. Chronic alcohol intake is associated with defective gut motility, which can cause an elevated level of endotoxin (lipopolysaccharide, LPS) in the liver^4^. Some studies have demonstrated a direct relationship between blood levels of endotoxin and the severity of liver fibrosis^5^,^6^. In addition, liver fibrosis can lead to cirrhosis, septae/nodules formation and portal hypertension^7^. Although, some significant progresses have been made in understanding ALF, the methods to treat this disease are still ineffective. Thus, the works focusing on novel and effective treatment methods to reverse alcohol-induced liver fibrosis are important.

In case of liver fibrosis, Kupffer cells (KCs) can produce a variety of proinflammatory cytokines to provoke hepatic stellate cells (HSCs) activation and hepatic injury, in which inflammation may be a bridge between liver injury and fibrosis^8^. At present, one specific KCs marker, CD68, has been widely used to monitor KCs activation^9^. HSCs are the major effector cells in the development of hepatic fibrosis^10^. In response to liver injury, quiescent HSCs undergo notable phenotypic alterations, including enhanced cell proliferation, expression of α-smooth muscle actin (α-SMA), and overproduction of extracellular matrix (ECM)^11^. Following a fibrogenic stimulus, HSCs lose their retinoid stores, proliferate and express excess α-SMA, and produce large amounts of ECM, including type I collagen (CoL1A1). HSCs are not only the main producers of ECM in the fibrotic liver, but also the targets of proinflammatory mediators^12^,^13^. Thus, HSCs activation is a critical event in hepatic fibrogenesis, and numerous attempts have been made to develop new strategies for the treatment of hepatic fibrosis^14^.

Although inflammation happens in all patients with hepatic fibrosis and correlates with fibrosis progression^12^, the molecular link between hepatic inflammation and fibrogenesis remains elusive. Toll-like receptors (TLRs) constitute a highly conserved family of receptors that recognise pathogen-associated molecular patterns and allow the host to detect microbial infection. TLRs can regulate innate and adaptive immune response^15^, which are also associated with noninfectious inflammatory diseases of liver^16^. TLR4, one receptor for LPS, can trigger two different signaling pathways, in which one is a myeloid differentiation factor 88 (MyD88)-dependent pathway and leads to the rapid activation of nuclear factor κB (NF-κB) and increased tumour necrosis factor-α (TNF-α) production, and the other is an MyD88-independent pathway requiring the Toll/interleukin-1 receptor (TIR)-containing adaptor molecule. Both pathways can stimulate downstream signal cascades, inducing the production of proinflammatory cytokines, chemokines, and type I interferon^17^.

NF-κB, a ubiquitous transcription factor, can govern the expression of certain proinflammatory and immune system-modulated genes^18^. NF-κB activation has been demonstrated with the development and survival of the activated HSCs^19^,^20^. One mechanism for this phenomenon is the response triggered by LPS signaling through TLR4. In brief, TLR4 activation in KCs and HSCs can cause the release of inflammatory cytokines and chemokines including interleukin (IL)-1, IL-6 and TNF-α as well as profibrogenic cytokines including transforming growth factor-b1 (TGF-β1). TGF-β1, a key activator of HSCs, can up-regulate the syntheses of some proteins associated with ECM and the cellular receptors of several matrix proteins^21^,^22^ to further promote hepatocyte injury and death as well as the deposition of ECM components in liver^16^.

Although many efforts have been made to investigate the underlying mechanisms of ALF, the effective treatment methods remain controversial and uncertain. Therefore, it is urgent to develop new drugs with high efficiency and low side-effect for the treatment of ALF. Herbal medicines have been used to treat hepatic fibrosis for a long time, and many natural products including sauchinone and helenalin show potent effects against hepatic fibrosis^23^,^24^. Thus, it is reasonable to explore new and effective natural products from medicinal herbs for the treatment of ALF.

Dioscin (Dio, shown in Supplemental Fig. 1), a natural steroid saponin, is widely present in some medicinal herbs^25^. Pharmacological studies have demonstrated that dioscin has anti-tumour and anti-hyperlipidaemic^26^,^27^ activities. In our previous studies, the protective effects of dioscin against carbon tetrachloride (CCl_4_)-and paracetamol-induced acute liver damages^28^,^29^ have been reported, and the actions of this compound against non-alcoholic fatty liver disease (NAFLD) and hepatic ischemia-reperfusion injury in rats have also been reported^30^,^31^. Furthermore, we also found that dioscin shows significantly protective effects against ethanol-induced liver injury^32^. However, to the best of our knowledge, the effects and molecular mechanisms of dioscin against ALF remain unknown.

Therefore, the aim of the present paper was to investigate the effects and possible mechanisms of dioscin against ALF.

## Results

### Dioscin inhibits proliferation of HSCs treated with LPS

**Figure 1:**
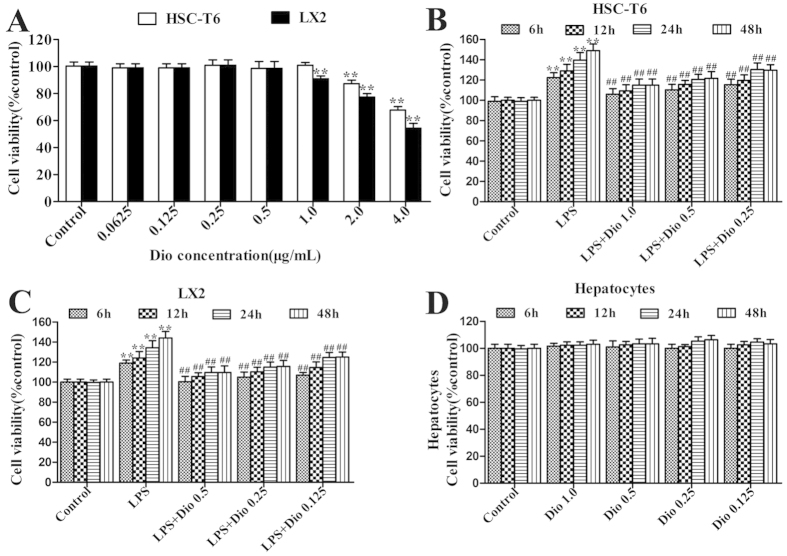
Dioscin inhibits HSCs proliferation induced by LPS. (**A**) Effects of dioscin on cell viability by MTT assay. (**B,C**) Effects of dioscin on the proliferation of cells treated with LPS. (**D**) Effects of dioscin on the cell viability of hepatocytes cells. The values are presented as the mean ± S.D. from triplicate wells. *p < 0.05 and **p < 0.01 vs. control group, ^#^p < 0.05 and ^##^p < 0.01 vs. model group.

As shown in Fig. 1A, dioscin significantly inhibited cell proliferation, and the compound at the concentrations of 0.5, 0.25 and 0.125 μg/ml for LX2 cells, and 1.0, 0.5 and 0.25 μg/ml for HSC-T6 cells was optimized. Under these conditions, dioscin effectively inhibited the proliferation of the HSCs caused by LPS with time- and dose-dependent manners (Fig. 1B,C). Importantly, dioscin at the concentrations of 1.0, 0.5, 0.25 and 0.125 μg/ml was not toxic to the isolated mouse primary hepatocytes (Fig. 1D).

### Dioscin rehabilitates alcohol-induced liver fibrosis and injury in mice

**Figure 2:**
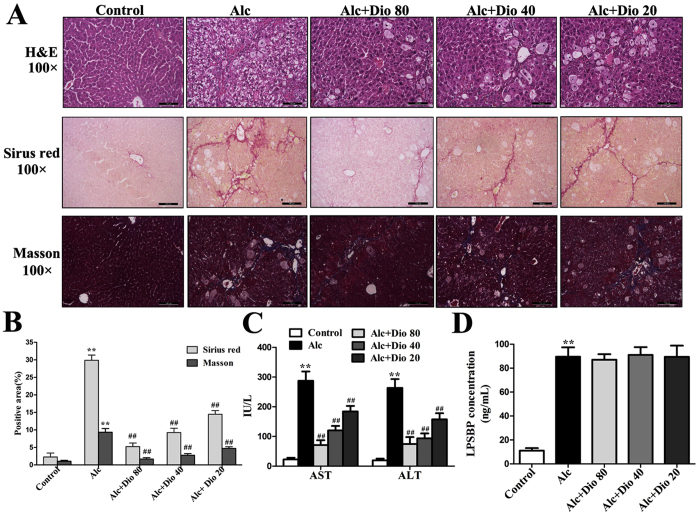
Dioscin ameliorates ALF in mice. (**A,B**) Liver fibrosis as assessed by H&E, Sirius red and Masson staining (100× original magnification). (**C**) Effects of dioscin on serum AST and ALT activities in mice treated with alcoholic liquid diet for 14 weeks. (**D**) Effects of dioscin on serum LPSBP levels in mice treated with alcoholic liquid diet for 14 weeks. The values are expressed as the mean ± SD (n = 8). *p < 0.05 and **p < 0.01 vs. control group, ^#^p < 0.05 and ^##^p < 0.01 vs. model group.

As shown in Fig. 2A, the livers from the normal group had an intact lobular architecture with clear central veins and radiating hepatic cords, whereas the livers from the alcohol- treated group exhibited extensive and severe haemorrhagic necrosis, destruction of liver architecture, inflammatory cell infiltration, and obvious hepatic collagen deposition. The mice in dioscin-treated (80, 40 and 20 mg/kg) groups showed significant reduction in hepatic fibrosis from 30.5% to 4.3%, 9.6%, and 14.7% by Sirius red staining, and also reduced the fibrosis from 9.7% in model group to 1.87%, 2.89% and 4.42% by Masson staining (Fig. 2B). These data indicated that dioscin attenuated alcohol-induced liver fibrosis and injury in mice. As shown in Fig. 2C, the increased AST and ALT levels were significantly attenuated by dioscin compared with model group. As shown in Fig. 2D, the level of LPSBP, a surrogate marker of LPS-TLR4 activation^33^, was notably increased in model mice, and dioscin treatment did not lead to a significant decrease.

### Dioscin attenuates inflammation and fibrosis *in vivo*

**Figure 3:**
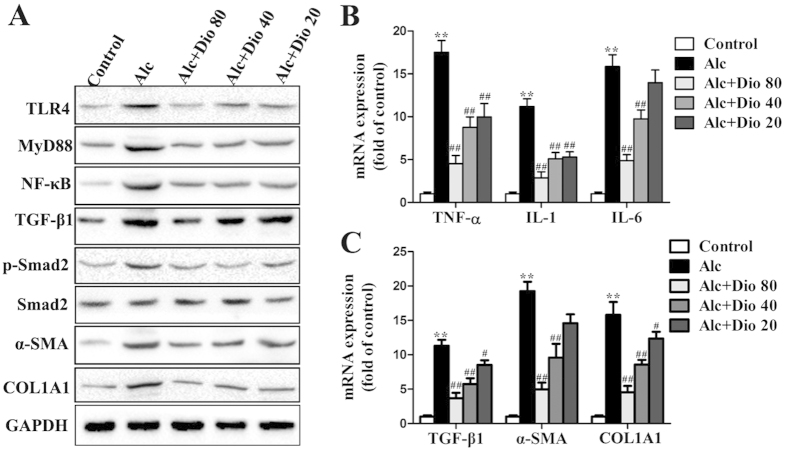
Dioscin down-regulates inflammation and fibrosis related mRNA and proteins *in vivo*. (**A**) Effects of dioscin on the protein levels of TLR4, MyD88, NF-κB, TGF-β1, p-Smad2/Smad2, α-SMA and COL1A1 in mice. The cropped gels are used and full-length gels are presented in Supplementary Fig. S8. (**B**) Effects of dioscin on the mRNA levels of TNF-α, IL-1 and IL-6 in mice. (**C**) Dioscin suppressed the mRNA levels of TGF-β1, α-SMA and COL1A1 following HSCs activation in mice. The values are expressed as the mean ± SD (n = 3). *p < 0.05 and **p < 0.01 vs. control group, ^#^p < 0.05 and ^##^p < 0.01 vs. model group.

As shown in Fig. 3A, the expression levels of TLR4, MyD88, NF-κB, TGF-β1, p-Smad2, α-SMA and COL1A1 in model group were markedly increased compared with normal mice, which were all significantly down-regulated by dioscin. The results of statistical analysis are provided in Supplemental Fig. 2. The livers from the mice in model group exhibited drastically increased hepatic mRNA levels of TNF-α, IL-1 and IL-6, which were all significantly decreased to 3.62-, 3.35- and 3.14-fold by 80 mg/kg of dioscin (Fig. 3B). In addition, the increased mRNA levels of TGF-β1, α-SMA and COL1A1 caused by alcohol were all significantly decreased to 3.13-, 4.12- and 4.07-f old, respectively, by 80 mg/kg of dioscin (Fig. 3C). Together, these data indicated that dioscin-attenuated liver fibrosis via down-regulating TLR4, not LPSBP.

### Dioscin inhibits kupffer cells and HSCs activations *in vivo*

**Figure 4:**
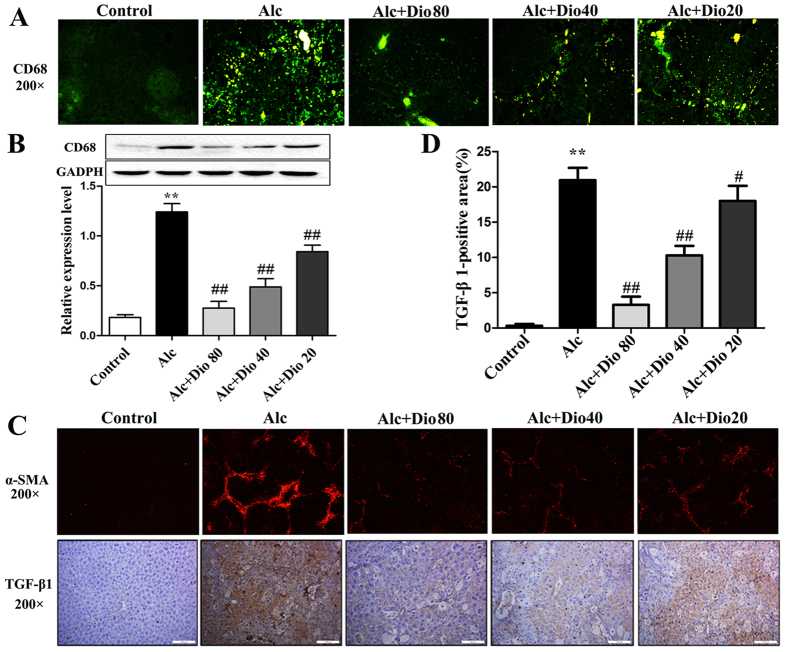
Dioscin attenuates HSCs and Kupffer cells activation *in vivo*. (**A,B**) Effects of dioscin on the expression levels of CD68 detected by immunofluorescence and western blotting assays. (**C,D**) Effects of dioscin on the expression levels of α-SMA and TGF-β1 in mice liver based on immunofluorescence and immunohisto -chemical assays (200 × original magnification). The values are expressed as the mean ± SD (n = 3). *p < 0.05 and **p < 0.01 vs. control group, ^#^p < 0.05 and ^##^p < 0.01 vs. model group.

As shown in Fig. 4A, the CD68^+^ KCs were obviously found in model group, which were significantly decreased by dioscin based on immunofluorescence assay, and the expression level of CD68 was also down-regulated by the compound based on western blotting assay (Fig. 4B). In addition, the effect of dioscin on HSCs activation *in vivo* was tested, and the results showed that the expression levels of fibronectin (shown in Supplemental Fig. 3), α-SMA and TGF-β1 (Fig. 4C,D) in the dioscin-treated mice were signifi -cantly decreased compared with those in the alcohol-treated mice. Furthermore, the p-Smad2 was obviously found in model group, which was significantly decreased by dioscin based on immunohistochemistry assay (shown in Supplemental Fig. 4).

### Dioscin reduces α-SMA and fibronectin levels in culture-activated HSCs

**Figure 5:**
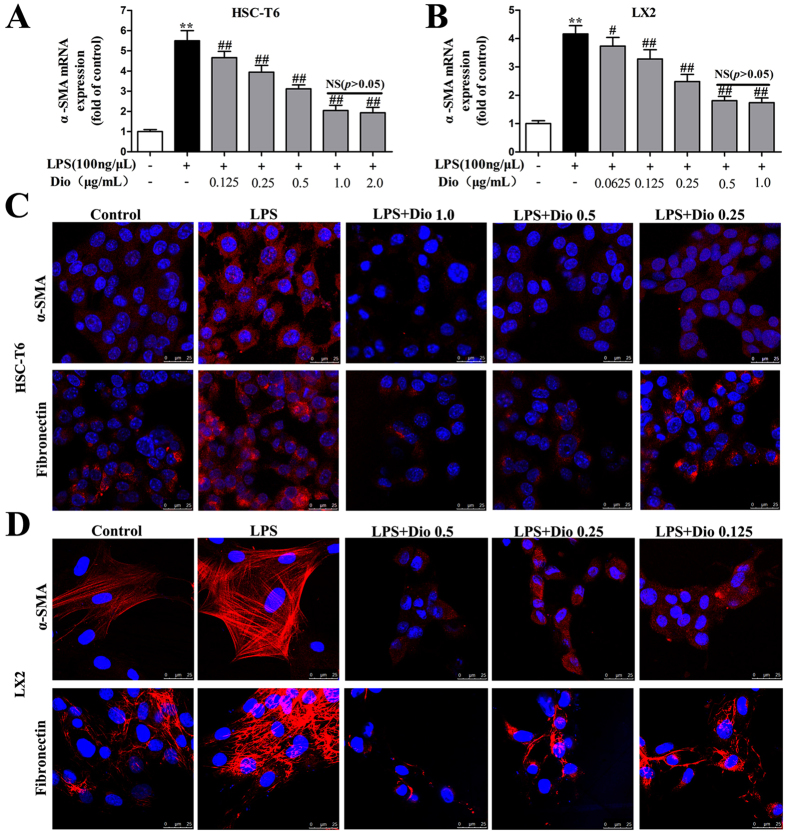
Dioscin reduces α-SMA and fibronectin levels in culture-activated HSCs. (**A,B**) Effects of dioscin on the mRNA levels of α-SMA in HSC-T6 and LX2 cells. (**C,D**) Effects of dioscin on α-SMA and fibronectin levels based on an immunofluorescence assay in HSC-T6 and LX2 cells (× 800 original magnification). The values are expressed as the mean ± SD (n = 3). *p < 0.05 and **p < 0.01 vs. control group, ^#^p < 0.05 and ^##^p < 0.01 vs. model group.

As shown in Fig. 5A,B, as expected, dioscin significantly reduced the mRNA level of α-SMA. However, dioscin at a concentration of over 1.0 μg/ml for HSC-T6 cells or 0.5 μg/ml for LX2 cells had no additional impact on α-SMA levels. Thus, dioscin at the concentration of 1.0 or 0.5 μg/ml to treat the culture-activated HSC-T6 and LX2 cells was used. As shown in Fig. 5C,D, the results showed that the levels of fibronectin and α-SMA were substantially decreased in HSC-T6 and LX2 cells by dioscin compared with the control groups based on immunofluorescence assays.

### Dioscin attenuates inflammation and inhibits HSCs activation *in vitro*

**Figure 6:**
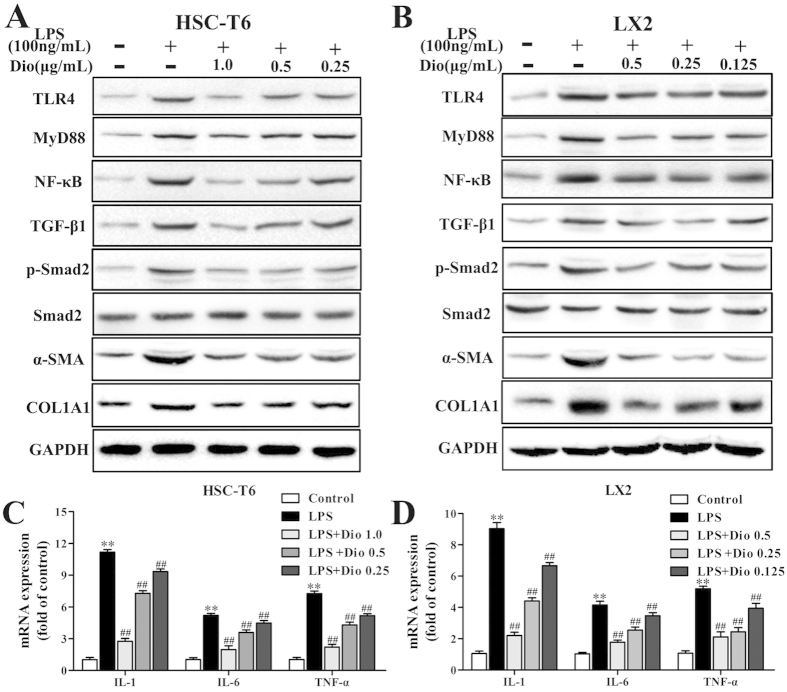
Dioscin attenuates inflammation and inhibits HSCs activation *in vitro*. (**A**,**B**) Effects of dioscin on the protein levels of TLR4, MyD88, NF-κB, TGF-β1, p-Smad2/ Smad2, α-SMA and COL1A1 in HSC-T6 and LX2 cells. The cropped gels are used and full-length gels are presented in Supplementary Fig. S9. (**C,D**) Effects of dioscin on the mRNA levels of IL-1, IL-6 and TNF-α in HSC-T6 and LX2 cells. The values are expressed as the mean ± SD (n = 3). *p < 0.05 and **p < 0.01 vs. control group, ^#^p < 0.05 and ^##^p < 0.01 vs. model group.

**Figure 7:**
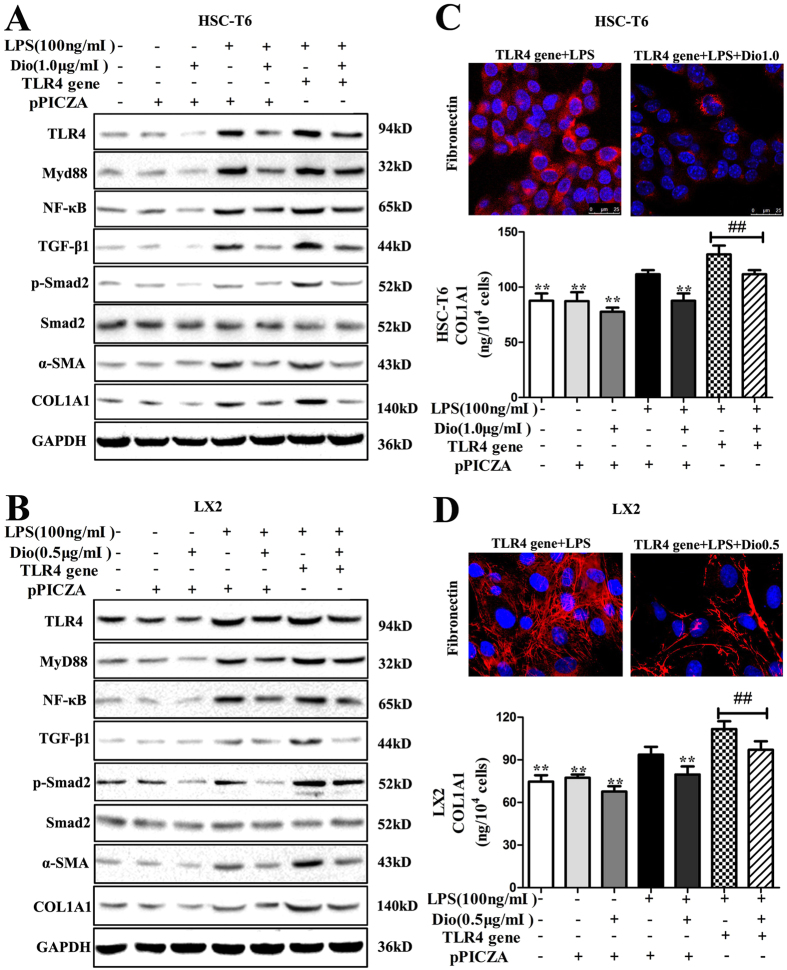
Dioscin attenuates inflammation and inhibits HSCs activation following TLR4 gene overexpression *in vitro*. (**A,B**) Effects of dioscin on the levels of TLR4, MyD88, NF-κB, TGF-β1, p-Smad2/ Smad2, α-SMA, and COL1A1 with or without the TLR4 gene or pPICZA in HSC-T6 and LX2 cells. The cropped gels are used and full-length gels are presented in Supplementary Fig. S10. (**C,D**) Effects of dioscin on fibronectin expression based on an immunofluorescence assay (×800 original magnification) and the effect on the level of secreted COL1A1 in supernatants measured by ELISA with TLR4 gene transfection in HSC-T6 and LX2 cells. The values are expressed as the mean ± SD (n = 3). *p < 0.05 and **p < 0.01 vs. model group; and ^**##**^p < 0.01 vs. LPS+TLR4 gene group.

As shown in Fig. 6A,B, the expression levels of TLR4, MyD88, NF-κB, TGF-β1, p-Smad2, α-SMA and COL1A1 in HSC-T6 and LX2 cells were significantly down-regulated by dioscin compared with the model groups. The results of statistical analysis are provided in Supplemental Fig. 5. In addition, the mRNA levels of TNF-α, IL-1 and IL-6 were all significantly decreased in HSC-T6 and LX2 cells treated with dioscin (Fig. 6C,D). With regard to the attenuating inflammation by dioscin, TLR4 gene transfection approach was used. As shown in Fig. 7A,B, TLR4 gene transfection weakened the inhibitory effect of MyD88 by dioscin (Supplemental Fig. 6). Similar results were also found for fibronectin and COL1A1 levels after TLR4 gene transfection (Fig. 7C,D).

### MyD88 and NF-κB mediate the inhibitory effect of HSCs activation by dioscin

**Figure 8:**
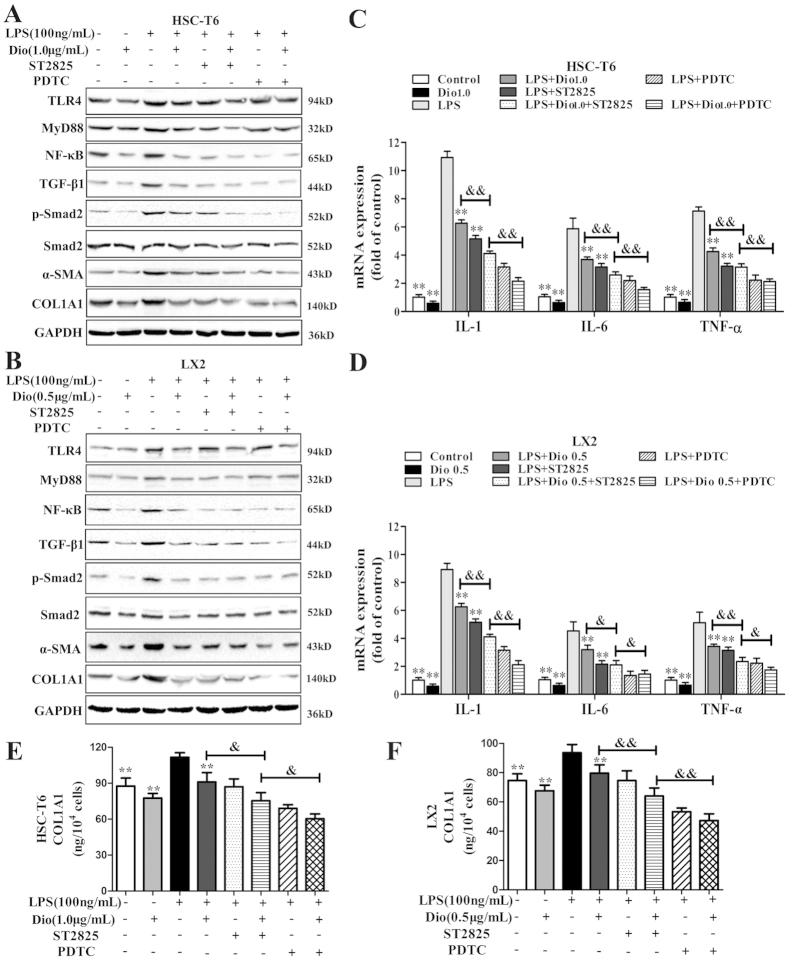
Both MyD88 and NF-κB are involved in inhibition of HSCs activation by dioscin. (**A,B**) Effects of dioscin on the levels of TLR4, MyD88, NF-κB, TGF-β1, p-Smad2/ Smad2, α-SMA and COL1A1 with or without ST2825 and PDTC in HSC-T6 and LX2 cells. The cropped gels are used and full-length gels are presented in Supplementary Fig. S11. (**C,D**) Effects of dioscin on the mRNA levels of IL-1, IL-6 and TNF-α measured by quantitative real-time PCR assay in HSC-T6 and LX2 cells. (**E,F**) Effects of dioscin on the level of secreted COL1A1 in supernatants measured by ELISA in HSC-T6 and LX2 cells. The values are expressed as the mean ± SD (n = 3). *p < 0.05 and**p < 0.01 vs. model group; and ^**&**^p < 0.05 and ^**&&**^p < 0.01 vs. LPS + Dio + ST2825 group.

As shown in Fig. 8A–D, treatment of HSCs with ST2825 (20 μM) for 2 h significantly inhibited MyD88 expression, whereas PDTC had no significant effect on the level of TLR4 or MyD88 in the culture-activated HSCs. In addition, the increased levels of MyD88, NF-κB, TNF-α, IL-1 and IL-6 caused by LPS were partially abolished by pre-treatment with ST2825. Likewise, both ST2825 and PDTC abolished the activation of HSCs by LPS, as demonstrated by the expression levels of TGF-β1, p-Smad2, α-SMA and COL1A1. ST2825, PDTC and dioscin decreased the expression level of NF-κB. No obvious changes in TLR4 level were noted in the culture- activated HSCs treated with ST2825 or PDTC. The results of statistical analysis are provided in Supplemental Fig. 7. As shown in Fig. 8E,F, treatment with ST2825 or PDTC partially reversed the production of secreted collagen elicited by LPS. These results showed that the MyD88-dependent inhibitory effect of HSCs activation by dioscin might be associated with the NF-κB pathway.

## Discussion

Alcohol abuse, one of the major causes of liver fibrosis, is sharply increasing worldwide^34^. ALF, a result of alcoholic liver injury, is characterised by the excessive accumulation of ECM in the liver. In contrast with the traditional view, ALF is a passive and irreversible pathological process induced by the necrosis of liver parenchymal cells, and recent evidences have shown that even advanced fibrosis is reversible^35^,^36^. Thus, development of novel and effective treatment method to reverse ALF is critical important. In the present work, dioscin showed significant effects against ALF as evidenced by the decreased AST and ALT levels, and the alleviation of histopathological changes. Furthermore, dioscin was effective in suppressing the TLR4/MyD88/NF-κB signaling pathway to inhibit HSCs activation and reduce ECM accumulation for attenuating liver fibrosis *in vivo* and *in vitro*.

Chronic hepatic inflammation is tightly linked to fibrosis in liver disease. Whereas chronic activation of inflammatory pathway has been shown to promote hepatocarc -inogenesis^37^,^38^, the molecular link between inflammation and hepatic fibrogenesis remains elusive. Liver is a main target of intestinally derived bacterial product, and the rate of bacterial translocation is increased in various models of hepatic diseases^39^. Here, we demonstrated that TLR4-mediated myofibroblast activation and fibrogenesis in liver, and TLR4-dependent modulation of TGF-β1 signaling provided a link between proinflammatory and profibrogenic signals.

It is well known that LPS/TLR4 signaling is critically involved in HSCs transactiv -ation during liver injury^40^. In addition, the increased gut permeability results in the increased LPS levels during ALD, and chronic liver injury and inflammation can promote HSCs activation and cause liver fibrosis^12^. Proinflammatory cytokines including interleukins (IL-1 and IL-6) and TNF-α are produced by inflammatory cells, and their levels are strictly regulated by proinflammatory and anti-inflammatory responses^41^. In these processes, NF-κB plays an important role in the regulation of inflammatory responses^42^,^43^. NF-κB activation can affect the levels of various proinflammatory cytokines including IL-1, IL-6 and TNF-α, and the profibrogenic factors including TGF-β1, which can enhance the survival and proliferation of activated HSCs^44^. In the present work, the antinflammatory capability of dioscin mainly resulted from decreased levels of MyD88, NF-κB, IL-1, IL-6 and TNF-α via the reduction of TLR4 expression. MyD88 played a role in the inhibitory effect of HSCs activation by dioscin, including reducing the expression levels of TGF-β1, α-SMA, COL1A1, fibronectin, and suppressing COL1A1 production. Thus, NF-κB pathway was involved in dioscin-mediated inhibition of HSCs activation *in vivo* and *in vitro*.

During the fibrogenesis development, many pathological factors including inflammation derived from KCs, angiogenesis and HSCs activation can lead to collagen deposition^45^. KCs or resident hepatic macrophages play an important role in modulating inflammation in liver fibrosis^46^. HSCs are the key effectors in the pathogenesis of liver fibrosis^47^. Therefore, the activated HSCs are considered as a major target for the attenuation or reversal of liver fibrosis^48^. Our results indicated that dioscin remarkably suppressed KCs, which was demonstrated by the decreased levels of CD68, and also suppressed HSCs activation *in vitro* and *in vivo* in association with reduced levels of TGF-β1, p-Smad2, COL1A1, α-SMA and fibronectin. In addition, primary mouse hepatocytes were not affected by dioscin under the used concentrations in this study. Thus, dioscin was not toxic to the hepatic parenchymal cells.

TLR4 has a critical role in alcohol-induced liver damage through inflammatory cytokine induction, independent of the common TLR adapter, MyD88 expression^49^,^50^. The results of this study showed that dioscin partially inhibited MyD88 level but did not completely abolish the inhibition of secreted collagen production. In addition, MyD88 was essential, but not sufficient, for the suppression of secreted collagen production and one or more factors in addition to MyD88 were also involved in this process. These data demonstrated that the decreased TLR4 expression by dioscin may underlie the decreased MyD88 expression. To clearly demonstrate whether TLR4 gene overexpression can affect COL1A1 production, we measured the levels of COL1A1 in HSC-T6 and LX2 cells following TLR4 overexpression, and the results suggested that the alterations in COL1A1 levels by dioscin may be mediated by TLR4.

It has been reported that NF-κB pathway plays an important role in regulating fibrogenic responses in liver, which is consistent with the recent report of decreased hepatic fibrogenesis after NF-κB inhibition^51^. In this study, we demonstrated that NF-κB signaling regulated the COL1A1 level in culture-activated HSCs. The level of COL1A1 still remained after PDTC inhibition, suggesting that other signaling pathways may also be involved. Importantly, the present study indicated that NF-κB served as a molecular link between inflammatory signal and HSCs activation. PDTC partially blocked the increased levels of TGF-β1, α-SMA and COL1A1, but PDTC alone had only a negligible effect on MyD88, suggesting that MyD88 was located upstream of NF-κB, and regulated collagen expression through NF-κB in culture- activated HSCs.

**Figure 9:**
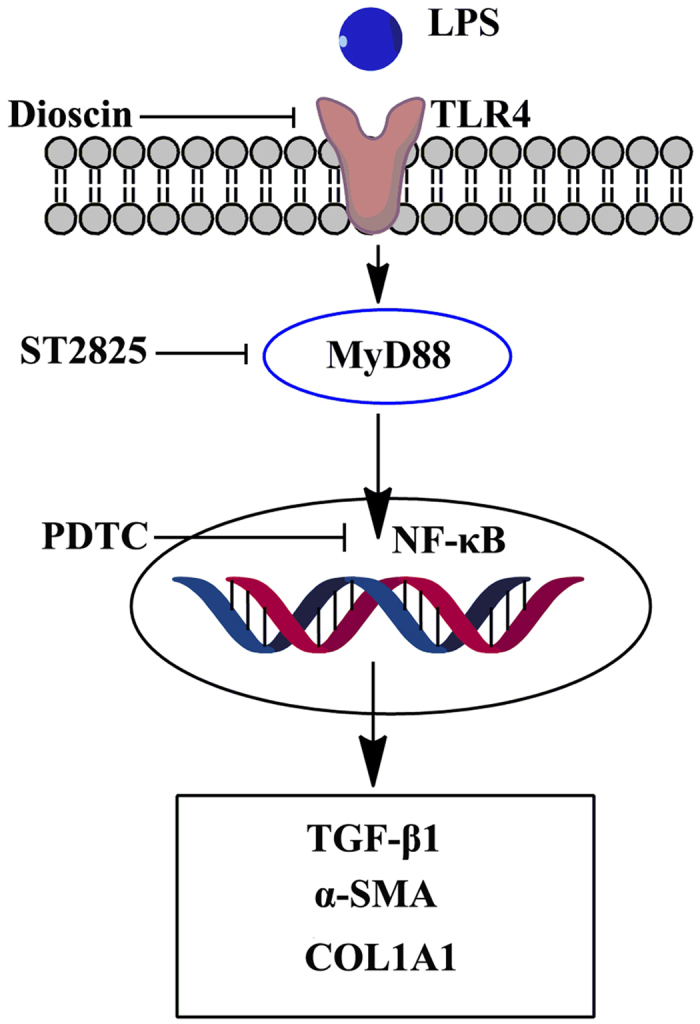
Schematic diagram of the anti-inflammatory effect of dioscin in inhibition of HSCs activation. Dioscin decreased the level of intracellular MyD88 by down-regulating the expression of TLR4, leading to the attenuation of inflamma- tion, which in turn, blocked the activation of NF-κB, resulting in the inhibition of HSCs activation.

In conclusion, based on our observations, a simplified pathway to describe the possible involvement of TLR4/MyD88/NF-κB signaling pathway in the inhibition of HSCs activation caused by dioscin *in vivo* and *in vitro* was found (Fig. 9). Dioscin decreased the MyD88 level via down-regulating TLR4 expression. Inhibition of NF-κB leaded to the inhibition of HSCs activation. Notably, the underlying mechanisms are certainly more complex than what is described here. In addition, our results do not exclude the possible involvement of other signaling pathways and mechanisms caused by dioscin to suppress ALD. These findings provide novel insights into the mechanisms of dioscin as a potent antifibrotic agent that may be used to treat ALF. However, at the present time, dioscin has only been used as one raw material to treat liver fibrosis in animals or cells, and is not suitable for directly used in clinical settings. Thus, the clinical application and data to support the present findings of the natural product are necessary in the future.

## Materials and Methods

### Tested drug

Dioscin was isolated from *Dioscorea nipponica* Makino in our laboratory with a purity of over 98%, and was analysed by high-performance liquid chromatography, and the chemical structure of the compound was identified by mass spectrometry and nuclear magnetic resonance^25^,^52^. Dioscin was dissolved in 0.5% carboxymethylce -llulose sodium (CMC-Na), which was freshly prepared each day and administered intragastrically (i.g.) to the mice at doses of 80, 40 and 20 mg/kg once daily according to our previous study^32^.

### Chemicals and reagents

Pyrrolidine dithiocarbamate (PDTC) and ST2825 were purchased from Sigma- Aldrich (St. Louis, MO, USA). Alanine aminotransferase (ALT) and aspartate amino -transferase (AST) kits were obtained from the Nanjing Jiancheng Institute of Biotechnology (Nanjing, China). A tissue protein extraction kit was obtained from Keygen Biotech. Co., Ltd. (Nanjing, China). Bicinchoninic acid (BCA) protein assay kit was purchased from the Beyotime Institute of Biotechnology (Jiangsu, China). A 3, 3′-diaminobenzidine (DAB) substrate kit was purchased from Zhong- shan Golden Bridge Biotechnology (Beijing, China); 4’, 6′-diamidino-2-phenylindole (DAPI), tris (hydroxymethyl) aminomethane (Tris), sodium dodecyl sulphate (SDS) and CMC-Na were purchased from Sigma. RNAiso Plus, a PrimeScript® RT Reagent Kit with gDNA Eraser (Perfect Real Time) and SYBR® Premix Ex Taq™ II (Tli RNase H Plus) were purchased from TaKaRa Biotechnology Co., Ltd. (Dalian, China).

### Animal models and experimental protocol

Five-week-old male C57BL/6J mice were purchased from the Experimental Animal Centre of Dalian Medical University, Dalian, China (quality certificate number: SCXK (Liao) 2008–0002). After 1 week of acclimatisation, the mice were randomly divided into five groups (n = 8 per group) as follows: control; alcoholic (Alc); and dioscin-treated (20, 40 and 80 mg/kg) groups, including Alc + Dio 80, Alc + Dio 40 and Alc + Dio 20. The mice were fed a commercially available liquid diet, according to Lieber and DeCarli28 (BioServ, Frenchtown, NJ, USA), which contained either ethanol (gradually increased to a final alcohol dose of 32 g/kg/day, equivalent to 36% of caloric intake) or maltose-dextrin (pair-fed group). After 14 weeks of feeding on the control or alcohol diet, the mice were sacrificed after an overnight fast. Then, blood and liver tissue were collected and stored for further analysis. All animals were housed in a controlled environment at 23 ± 2 °C under a 12-h dark/light cycle with free access to food and water. All experimental procedures were approved by the Animal Care and Use Committee of Dalian Medical University and performed in strict accordance with the People’s Republic of China Legislation Regarding the Use and Care of Laboratory Animals.

### Assessments of biochemical parameters

The serum activities of AST and ALT were detected using detection kits based on the manufacturer’s instructions. The serum level of LPS-binding protein (LPSBP) was determined using a sandwich enzyme-linked immunosorbent assay (ELISA; Abnova Corporation, Taiwan, China), as recommended by the manufacturer. All assays were performed in triplicate.

### Histological and immunohistochemical assays

Liver tissues were fixed in 10% formalin and embedded in paraffin. Five-micron- thick sections were stained with haematoxylin-eosin (H&E), Masson and Sirius red stains. Images were acquired by light microscopy (Nikon Eclipse TE2000-U, Nikon, Japan), and the degree of liver fibrosis was quantified using Image-Pro Plus 6.0 software. For immunohistochemical staining, the paraffin sections were incubated with an anti-TGF-β1 and p-Smad2 antibodies and a biotinylated secondary antibody, followed by incubation with an avidin-biotin-peroxidase complex. Then, the signals were visualised using DAB. Images were acquired by light microscopy (Nikon Eclipse TE2000-U, Nikon, Japan).

### Cell culture

Immortalised rat HSC-T6 and human LX2 cell lines with the characteristics of an activated HSCs phenotype were purchased from the Cell Bank of the Xiangya Central Experiment Laboratory of Central South University (Changsha, China) and the Cancer Institute and Hospital of the Chinese Academy of Medical Sciences, Beijing, China. The cells were cultured in Dulbecco’s Modified Eagle’s Medium (Gibco, Carlsbad, CA, USA) supplemented with 10% FBS and antibiotics (100 IU/ml penicillin and 100 mg/ml streptomycin) in humidified air containing 5% CO_2_ at 37 °C. HSC-T6 and LX2 cells were pretreated with different concentrations of dioscin (1, 0.5 and 0.25 μg/ml for HSC-T6; and 0.5, 0.25 and 0.125 μg/ml for LX2) and challenged with LPS (100 ng/ml) for 24 h.

### Cell toxicity assay

Hepatocyte cells were isolated from male C57BL/6J mice (20 ± 2 g) and cultured as previously described^53^,^54^. The isolated mouse primary hepatocyte cells were plated into 96-well plates at a density of 5 × 10^4^ cells/ml per well for 24 h before treatment and then incubated for another 24 h in the presence of different concentrations of dioscin (1, 0.5 and 0.25 μg/ml). The cell toxicity caused by dioscin was measured using the MTT method.

### Immunofluorescence assay of α-SMA and fibronectin in cells and liver

The HSC-T6 and LX2 cells were plated into 12-well plates (2 × 10^5^ cells/well) with aseptic coverslips, pretreated with different concentrations of dioscin (1, 0.5 and 0.25 μg/ml for HSC-T6; and 0.5, 0.25 and 0.125 μg/ml for LX2) and challenged with LPS (100 ng/ml) for 24 h. After incubation, the cells were washed with PBS. Then, they were fixed in 4% formaldehyde in PBS for 10 min and permeabilised with 0.2% Triton-X 100 in PBS for 10 min at room temperature. The cells were incubated with 2% BSA for the blocking of nonspecific binding sites. The paraffin sections from the animals were deparaffinised with xylene (two times for 15 min each) and rehydrated with different concentrations of alcohol (100, 90, 80, 70 and 60%) for 5 min. After the sections were incubated in 3% H_2_O_2_ in PBS for 30 min, nonspecific protein binding was blocked using normal goat serum for 30 min. A primary antibody against CD68 (1: 100 dilution), α-SMA (1: 100 dilution) or fibronectin (1: 100 dilution) was incubated with the fixed cells and the formalin-fixed and deparaffinised liver tissue sections overnight at 4 °C, and then the cells and liver tissue sections were incubated with a fluorescein-labelled secondary antibody for 1 h. Eventually, cell nuclei were stained with DAPI (5 μg/ml). All samples were imaged using a laser scanning confocal microscope (Leica, TCS SP5, Germany).

### COL1A1 ELISA

The level of secreted COL1A1 in the culture media was measured using a Rat Col I EIA Kit (R&D Systems, Minneapolis, MN, USA) according to the manufacturer’s instructions.

### Inhibitors of MyD88 and NF-κB in cells

The HSC-T6 and LX2 cells were plated into 6-well plates (2 × 10^5^ cells/well), and then the cells were exposed to ST2825 (20 μM) or PDTC (100 μM)^55^ for 2 h. After incubation, the cells were pre-treated with dioscin (1.0 μg/ml for HSC-T6 cells and 0.5 μg/ml for LX2 cells) and challenged with LPS (100 ng/ml) for 24 h for further analysis.

### TLR4 gene transfection

The HSC-T6 and LX2 cells were transfected with pPICZA-TLR4 plasmid DNA using Lipofectamine Plus Reagent (Invitrogen Life Technologies, CA, USA) according to the manufacturer’s instructions. 24 h after transfection, the cells were subjected to serum deprivation for 24 h before exposure to LPS (100 ng/ml) in the presence or absence of dioscin (1.0 μg/ml for HSC-T6 and 0.5 μg/ml for LX2) for an additional 24 h.

### Quantitative real-time PCR assay

Total RNA samples from the culture-activated HSC-T6 and LX2 cells and mouse livers were extracted using RNAiso Plus reagent following the manufacturer’s protocol. Reverse transcription for cDNA synthesis and quantitative real-time PCR were performed as previously described^56^. The forward (F) and reverse (R) primers for the tested genes are listed in Supplemental Table 1. For each sample, the *Ct* values for the target gene and GAPDH (as a calibrator) were determined based on standard curves. The calculated relative *Ct* value of each gene was divided by the relative value of GAPDH. Then, the expression level of each gene in the control group was set to one-fold and used to determine the relative levels in the other samples (*n*-fold).

### Western blotting assay

Total cellular protein from the culture-activated HSC-T6 and LX2 cells and mouse livers were extracted following standard protocols (Beyotime Biotechnology, Haimen, China), and the protein content was determined using a BCA Protein Assay Kit from Beyotime Biotechnology. Proteins (50 μg/lane) were subjected to SDS- PAGE and then transferred to a nitrocellulose membrane. After blocking, the membranes were incubated for 1 h at room temperature or overnight at 4 °C. The primary antibodies are listed in Supplemental Table 2. The blots were then incubated with horseradish peroxidise-conjugated antibodies for 2 h at room temperature at a 1: 2000 dilution (Beyotime Institute of Biotechnology, China). Protein expression was detected by the enhanced chemiluminescence (ECL) method and imaged with a Bio-Spectrum Gel Imaging System (UVP, USA). To eliminate variations due to protein quantity and quality, the data were adjusted to GAPDH expression (IOD of objective protein versus IOD of GAPDH protein). However, the protein levels of p-Smad2/Smad2 were not adjusted to GADPH expression.

### Statistical analyses

All data were evaluated as the mean and standard deviation (SD). Statistical analysis of the quantitative data for multiple group comparisons was performed using one-way analysis of variance (ANOVA) followed by Duncan’s test, whereas paired comparisons were performed using the *t* test with SPSS software (ver. 20.0; SPSS, Chicago, IL, USA). The results were considered to be significant at p < 0.05.

## Additional Information

**How to cite this article**: Liu, M. *et al*. Dioscin alleviates alcoholic liver fibrosis by attenuating hepatic stellate cell activation via the TLR4/MyD88/NF-κB signaling pathway. *Sci. Rep*. **5**, 18038; doi: 10.1038/srep18038 (2015).

## Supplementary Material

Supplementary Information

## References

[b1] Yan A. W. & Schnabl B. Bacterial translocation and changes in the intestinal microbiome associated with alcoholic liver disease. World J Hepatol 4, 110–118 (2012). 22567183 10.4254/wjh.v4.i4.110 PMC3345535

[b2] Tsukamoto H. Conceptual importance of identifying alcoholic liver disease as a lifestyle disease. J Gastroentero 42, 603–609 (2007). 10.1007/s00535-007-2075-3 17701122

[b3] Tang Y., Forsyth C. B., Banan A., Fields J. Z. & Keshavarzian A. Oats supplem- entation prevents alcohol-induced gut leakiness in rats by preventing alcohol- induced oxidative tissue damage. J Pharmacol Exp Ther 329, 952–958 (2009). 19276402 10.1124/jpet.108.148643 PMC2683774

[b4] Tsukamoto H., Rippe R., Niemela O. & Lin M. Roles of oxidative stress in activation of Kupffer and Ito cells in liver fibrogenesis. J Gastroenterol Hepatol 10, S50–53 (1995). 8589343 10.1111/j.1440-1746.1995.tb01798.x

[b5] Iimuro Y. . Female rats exhibit greater susceptibility to early alcohol-induced liver injury than males. Am J Physiol 272, G1186–1194 (1997). 9176229 10.1152/ajpgi.1997.272.5.G1186

[b6] Rivera C. A., Tcharmtchi M. H., Mendoza L. & Smith C. W. Endotoxemia and hepatic injury in a rodent model of hindlimb unloading. J Appl Physiol 95, 1656– 1663 (2003). 12794033 10.1152/japplphysiol.00302.2003

[b7] Han Y. P. . Essential role of matrix metalloproteinases in interleukin-1- induced myofibroblastic activation of hepatic stellate cell in collagen. J Biol Chem 279, 4820–4828 (2004). 14617627 10.1074/jbc.M310999200 PMC2430939

[b8] Liu C. . Kupffer cells are associated with apoptosis, inflammation and fibrotic effects in hepatic fibrosis in rats. Lab Invest 90, 1805–1816 (2010). 20921949 10.1038/labinvest.2010.123

[b9] Buechler C., Ritter M., Orsó E., Langmann T., Kluchen J. & Schmitz G. Regulation of scavenger receptor CD163 expression in human monocytes and macrophages by pro- and anti-inflammatory stimuli. J Leukoc Biol 67, 97–103 (2000). 10648003

[b10] Gur C. . NKp46-mediated killing of human and mouse hepatic stellate cells attenuates liver fibrosis. Gut 61, 885–893 (2012). 22198715 10.1136/gutjnl-2011-301400

[b11] Van Hul N. K., Abarca-Quinones J., Sempoux C., Horsmans Y. & Leclercq I. A. Relation between liver progenitor cell expansion and extracellular matrix deposition in a CDE-induced murine model of chronic liver injury. Hepatology 49, 1625–1635 (2009). 19296469 10.1002/hep.22820

[b12] Bataller R. & Brenner D. A. Liver fibrosis. J Clin Invest 115, 209–218 (2005). 15690074 10.1172/JCI24282 PMC546435

[b13] Friedman S. L. Molecular regulation of hepatic fibrosis, an integrated cellular response to tissue injury. J Biol Chem 275, 2247–2250 (2000). 10644669 10.1074/jbc.275.4.2247

[b14] Friedman S. L. Liver fibrosis-from bench to bedside. J Hepatol 38, S38–53 (2003). 12591185 10.1016/s0168-8278(02)00429-4

[b15] Akira S. & Takeda K. Toll-like receptor signalling. Nat Rev Immunol 4, 499– 511, (2004). 15229469 10.1038/nri1391

[b16] Uesugi T., Froh M., Arteel G. E., Bradford B. U. & Thurman R. G. Toll-like receptor 4 is involved in the mechanism of early alcohol-induced liver injury in mice. Hepatology 34, 101–108 (2001). 11431739 10.1053/jhep.2001.25350

[b17] Kawai T. & Akira S. The role of pattern-recognition receptors in innate immunity: update on Toll-like receptors. Nat Immunol 11, 373–384 (2010). 20404851 10.1038/ni.1863

[b18] Karin M. & Lin A. NF-kappaB at the crossroads of life and death. Nat Immunol 3, 221–227 (2002). 11875461 10.1038/ni0302-221

[b19] Lang A., Schoonhoven R., Tuvia S., Brenner D. A. & Rippe R. A. Nuclear factor kappaB in proliferation, activation, and apoptosis in rat hepatic stellate cells. J Hepatol 33, 49–58 (2000). 10905586 10.1016/s0168-8278(00)80159-2

[b20] Kweon Y. O. . Gliotoxin-mediated apoptosis of activated human hepatic stellate cells. J Hepatol 39, 38–46 (2003). 12821042 10.1016/s0168-8278(03)00178-8

[b21] Hernandez-Gea V. & Friedman S. L. Pathogenesis of liver fibrosis. Annu Rev Pathol 6, 425–456 (2011). 21073339 10.1146/annurev-pathol-011110-130246

[b22] Marra F., Galastri S., Aleffi S. & Pinzani M. Stellate cells in: Dufour J. F., Clavien P. A. (Eds.), Signaling Pathways in Liver Diseases, Springer-Verlag, Berlin, Heidelberg, 41–68 (2010).

[b23] Lee J. H. . Sauchinone attenuates liver fibrosis and hepatic stellate cell activation through TGF-beta/Smad signaling pathway. Chem Biol Interact 224c, 58–67 (2014). 25451574 10.1016/j.cbi.2014.10.005

[b24] Lin X. . Helenalin attenuates alcohol-induced hepatic fibrosis by enhancing ethanol metabolism, inhibiting oxidative stress and suppressing HSC activation. Fitoterapia 95, 203–213 (2014). 24704336 10.1016/j.fitote.2014.03.020

[b25] Yin L. H. . An Economical Method for Isolation of Dioscin from *Dioscorea nipponica* Makino by HSCCC Coupled with ELSD, and a Computer-Aided UNIFAC Mathematical Model. Chromatographia 71, 15–23 (2010).

[b26] Hsieh M. J., Tsai T. L., Hsieh Y. S., Wang C. J. & Chiou H. L. Dioscin -induced autophagy mitigates cell apoptosis through modulation of PI3K/Akt and ERK and JNK signaling pathways in human lung cancer cell lines. Arch Toxicol 87, 1927–1937 (2013). 23552851 10.1007/s00204-013-1047-z PMC3824840

[b27] Li H. . Anti-thrombotic activity and chemical characterization of steroidal saponins from *Dioscorea zingiberensis* C.H. Wright. Fitoterapia 81, 1147–1156 (2010). 20659537 10.1016/j.fitote.2010.07.016

[b28] Lu B. . Mechanism investigation of dioscin against CCl_4_-induced acute liver damage in mice. Environ Toxicol Pharmacol 34, 127–135 (2012). 22516057 10.1016/j.etap.2012.03.010

[b29] Zhao X. . Dioscin, a natural steroid saponin, shows remarkable protective effect against acetaminophen-induced liver damage *in vitro* and *in vivo*. Toxicol Lett 214, 69–80 (2012). 22939915 10.1016/j.toxlet.2012.08.005

[b30] Xu L. . iTRAQ-based proteomics for studying the effects of dioscin against nonalcoholic fatty liver disease in rats. RSC Adv 4, 30704–30711 (2014).

[b31] Tao X. . Dioscin attenuates hepatic ischemia-reperfusion injury in rats through inhibition of oxidative-nitrative stress, inflammation and apoptosis. Transplantation 98, 604–611 (2014). 25083618 10.1097/TP.0000000000000262

[b32] Xu T. . Protective effects of dioscin against alcohol-induced liver injury. Arch Toxicol 88, 739–753 (2014). 24146112 10.1007/s00204-013-1148-8

[b33] Albillos A. . Increased lipopolysaccharide binding protein in cirrhotic patients with marked immune and hemodynamic derangement. Hepatology 37, 208–217 (2003). 12500206 10.1053/jhep.2003.50038

[b34] Bosetti C. . Worldwide mortality from cirrhosis: an update to 2002. J Hepatol 46, 827–839 (2007). 17336419 10.1016/j.jhep.2007.01.025

[b35] Safadi R. & Friedman S. L. Hepatic fibrosis–role of hepatic stellate cell activation. MedGenMed 4, 27 (2002). 12466770

[b36] Bonis P. A. L., Friedman S. L. & Kaplan M. M. Is Liver Fibrosis Reversible? New Engl J Med 344, 452–454 (2001). 11172184 10.1056/NEJM200102083440610

[b37] Maeda S., Kamata H., Luo J. L., Leffert H. & Karin M. IKKbeta couples hepatocyte death to cytokine-driven compensatory proliferation that promotes chemical hepatocarcinogenesis. Cell 121, 977–990 (2005). 15989949 10.1016/j.cell.2005.04.014

[b38] Pikarsky E. . NF-kappaB functions as a tumour promoter in inflammation -associated cancer. Nature 431, 461–466 (2004). 15329734 10.1038/nature02924

[b39] Seki E. . TLR4 enhances TGF-beta signaling and hepatic fibrosis. Nat Med 13, 1324–1332 (2007). 17952090 10.1038/nm1663

[b40] Ting B., Li-Hua L., Yan-Ling W., Ying W. & Ji-Xing N. Thymoquinone attenuates liver fibrosis via PI3K and TLR4 signaling pathways in activated hepatic stellate cells. Int Immunopharmacol 15, 275–281 (2013). 23318601 10.1016/j.intimp.2012.12.020

[b41] Knolle P. Liver Immunology (eds M. Eric Gershwin John M. Vierling & Michael P Manns) **Ch. 5**, 55–64 (2014).

[b42] Fox E. S., Kim J. C. & Tracy T. F. NF-kappaB activation and modulation in hepatic macrophages during cholestatic injury. J Surg Res 72, 129–134 (1997). 9356233 10.1006/jsre.1997.5172

[b43] Gabele E. . TNFalpha is required for cholestasis-induced liver fibrosis in the mouse. Biochem Biophys Res Commun 378, 348–353 (2009). 18996089 10.1016/j.bbrc.2008.10.155 PMC5052129

[b44] Ghazwani M. . Anti-fibrotic effect of thymoquinone on hepatic stellate cells. Phytomedicine 21, 254–260 (2014). 24182989 10.1016/j.phymed.2013.09.014

[b45] Hernandez-Gea V. & Friedman S. L. Pathogenesis of liver fibrosis. Annu Rev Pathol 28, 425–456 (2011). 21073339 10.1146/annurev-pathol-011110-130246

[b46] Minics S. D. . Gene expression profiles during hepatic stellate cell activation in culture and *in vivo*. Gastroenterology 132, 1937–1946 (2007). 17484886 10.1053/j.gastro.2007.02.033

[b47] Friedman S. L. Mechanisms of hepatic fibrogenesis. Gastroenterology 134, 1655–1669 (2008). 18471545 10.1053/j.gastro.2008.03.003 PMC2888539

[b48] Liu Y. . Inhibition of PDGF, TGF-beta, and Abl signaling and reduction of liver fibrosis by the small molecule Bcr-Abl tyrosine kinase antagonist Nilotinib. J Hepatol 55, 612–625 (2011). 21251937 10.1016/j.jhep.2010.11.035

[b49] Dey A. & Cederbaum A. I. Alcohol and oxidative liver injury. Hepatology 43, S63–S74 (2006). 16447273 10.1002/hep.20957

[b50] Arteel G. E. Oxidants and antioxidants in alcohol-induced liver disease. Gastroenterology 124, 778–790 (2003). 12612915 10.1053/gast.2003.50087

[b51] Oakley F. . Inhibition of inhibitor of kappaB kinases stimulates hepatic stellate cell apoptosis and accelerated recovery from rat liver fibrosis. Gastroenterology 128, 108–120 (2005). 15633128 10.1053/j.gastro.2004.10.003

[b52] Hu M. . Cytotoxicity of dioscin in human gastric carcinoma cells through death receptor and mitochondrial pathways. J Appl Toxicol 33, 712–722 (2013). 22334414 10.1002/jat.2715

[b53] Teratani T. . A high-cholesterol diet exacerbates liver fibrosis in mice via accumulation of free cholesterol in hepatic stellate cells. Gastroenterology 142, 152–164 (2012). 21995947 10.1053/j.gastro.2011.09.049

[b54] Schäfer S., Zerbe O. & Gressner A. M. The synthesis of proteoglycans in fat- storing cells of rat liver. Hepatology 7, 680–687 (1987). 3111965 10.1002/hep.1840070411

[b55] Shi H. . Effect of chlorogenic acid on LPS-induced proinflammatory signaling in hepatic stellate cells. Inflamm Res 62, 581–587 (2013). 23483217 10.1007/s00011-013-0610-7

[b56] Reilly M. P. & Rader D. J. The metabolic syndrome: more than the sum of its parts? Circulation 108, 1546–1551 (2003). 14517150 10.1161/01.CIR.0000088846.10655.E0

