# Author Correction: Dioscin alleviates alcoholic liver fibrosis by attenuating hepatic stellate cell activation via the TLR4/MyD88/NF-κB signaling pathway

**DOI:** 10.1038/s41598-020-74987-w

**Published:** 2020-10-22

**Authors:** Min Liu, Youwei Xu, Xu Han, Lianhong Yin, Lina Xu, Yan Qi, Yanyan Zhao, Kexin Liu, Jinyong Peng

**Affiliations:** College of Pharmacy, Dalian Medical University, No. 9 West Part of Lvshunnan Road, Dalian, 116044 China

Correction to: *Scientific Reports* 10.1038/srep18038, published online 10 December 2015

This Article contains an error.

In Figure 4C, the image is incorrect for the Alc+Dio80 panel. The correct Figure appears below as Figure 1.

**Figure 1:**
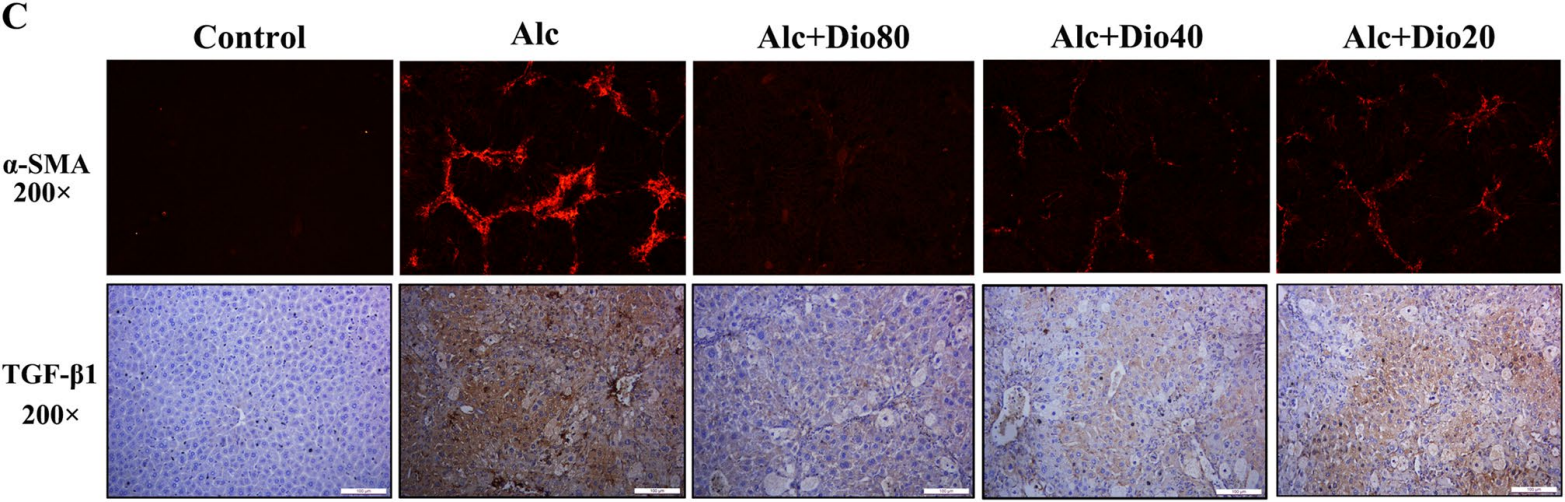
A corrected version of Figure 4C in the Article.

