# Supplementary material for: Dioscin alleviates alcoholic liver fibrosis by attenuating hepatic stellate cell activation via the TLR4/MyD88/NF-κB signaling pathway
Source: Sci Rep. 2015 Dec 10;5:18038. doi: 10.1038/srep18038 (PMC4674875; doi:10.1038/srep18038)
Supplement: Supplementary Information [file srep18038-s1.doc]

**Dioscin alleviates** **alcoholic liver fibrosis by attenuating hepatic stellate cell activation via the TLR4/MyD88/NF-κB signaling pathway**

Min Liu, Youwei Xu, Xu Han, Lianhong Yin, Lina Xu, Yan Qi, Yanyan Zhao, Kexin Liu, Jinyong Peng*

*College of Pharmacy, Dalian Medical University, No. 9 West Part of Lvshunnan Road,*

*Dalian 116044, China*

Corresponding author,

Dr. Jinyong Peng

College of Pharmacy

Dalian Medical University

Dalian, China

Tel.: +86 411 8611 0411

Fax: +86 411 8611 0411

Email: jinyongpeng2008@126.com

**Supplemental Table 1** The primer sequences used for real-time PCR assay in the present work.

| Gene | GenBank accession | Forward primer (5′-3′) | Reverse primer (5′-3′) |
| --- | --- | --- | --- |
| MouseGAPDH | NM_008084.2 | TGTGTCCGTCGTGGATCTGA | TTGCTGTTGAAGTCGCAGGAG |
| Mouse TNF-α | NM_013693.2 | TATGGCCCAGACCCTCACA | GGAGTAGACAAGGTACAACCCATC |
| Mouse IL1 | NM_008361.3 | TCCAGGATGAGGACATGAGCAC | GAACGTCACACACCAGCAGGTTA |
| Mouse IL6 | NM_031168.1 | CCACTTCACAAGTCGGAGGCTTA | CCAGTTTGGTAGCATCCATCATTTC |
| MouseTGF-β1 | NM_206958.2 | TCGGGTCACGGAAGATTCAG | GCAAGGGAAGATCTCACAGTTGT |
| Mouse α-SMA | NM_007392.3 | TGCCGAGCGTGAGATTGTC | CGTTCGTTTCCAATGGTGATC |
| MouseCOL1A1 | NM_007742.3 | TGACTGGAAGAGCGGAGAGTACT | TTCGGGCTGATGTACCAGTTC |
| RatGAPDH | NM_017008.3 | GGCACAGTCAAGGCTGAGAATG | ATGGTGGTGAAGACGCCAGTA |
| Rat IL1-β | NM_031512.2 | CCCTGAACTCAACTGTGAAATAGCA | CCCAAGTCAAGGGCTTGGAA |
| Rat IL-6 | NM_012589.1 | ATTGTATGAACAGCGATGATGCAC | CCAGGTAGAAACGGAACTCCAGA |
| Rat TNF-α | NM_012675.3 | TCAGTTCCATGGCCCAGAC | GTTGTCTTTGAGATCCATGCCATT |
| HumanGAPDH | NM_002046.3 | GCACCGTCAAGGCTGAGAAC | TGGTGAAGACGCCAGTGGA |
| Human IL-1 | NM_000576.2 | CTGAGCACCTTCTTTCCCTTCA | TGGACCAGACATCACCAAGCT |
| Human IL-6 | NM_000600.3 | TGGCTGAAAAAGATGGATGCT | TCTGCACAGCTCTGGCTTGT |
| HumanTNF-α | NM_000594.3 | TGTAGCCCATGTTGTAGCAAACC | GAGGACCTGGGAGTAGATGAGGTA |

**Supplemental Table 2** The information of the antibodies used in the present work

for western blotting assay

| Antibody | Source | Dilutions | Company |
| --- | --- | --- | --- |
| GAPDH | Rabbit | 1:2000 | Proteintech Group, Chicago, USA |
| TLR4 | Rabbit | 1:1000 | Proteintech Group, Chicago, USA |
| MyD88 | Rabbit | 1:1000 | Proteintech Group, Chicago, USA |
| NF-κB | Rabbit | 1:1000 | Proteintech Group, Chicago, USA |
| TGF-β1 | Rabbit | 1:1000 | Proteintech Group, Chicago, USA |
| p-Smad2 | Rabbit | 1:1000 | Proteintech Group, Chicago, USA |
| Smad2 | Rabbit | 1:1000 | Proteintech Group, Chicago, USA |
| α-SMA | Rabbit | 1:1000 | Proteintech Group, Chicago, USA |
| COL1A1  CD68 | Rabbit  Rabbit | 1:1000  1:1000 | Proteintech Group, Chicago, USA Proteintech Group, Chicago, USA |


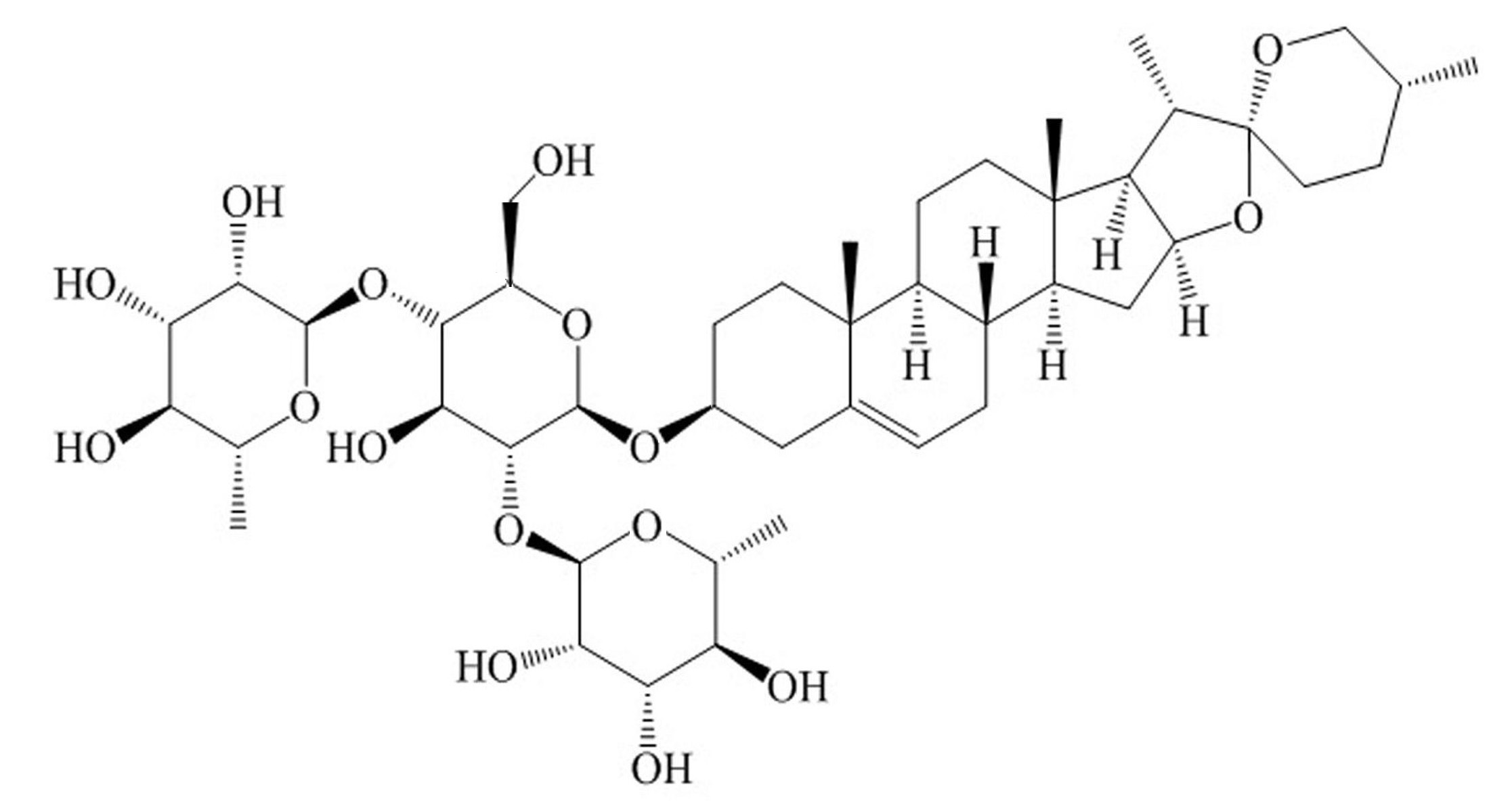


Supplemental Figure 1. The chemical structure of dioscin.


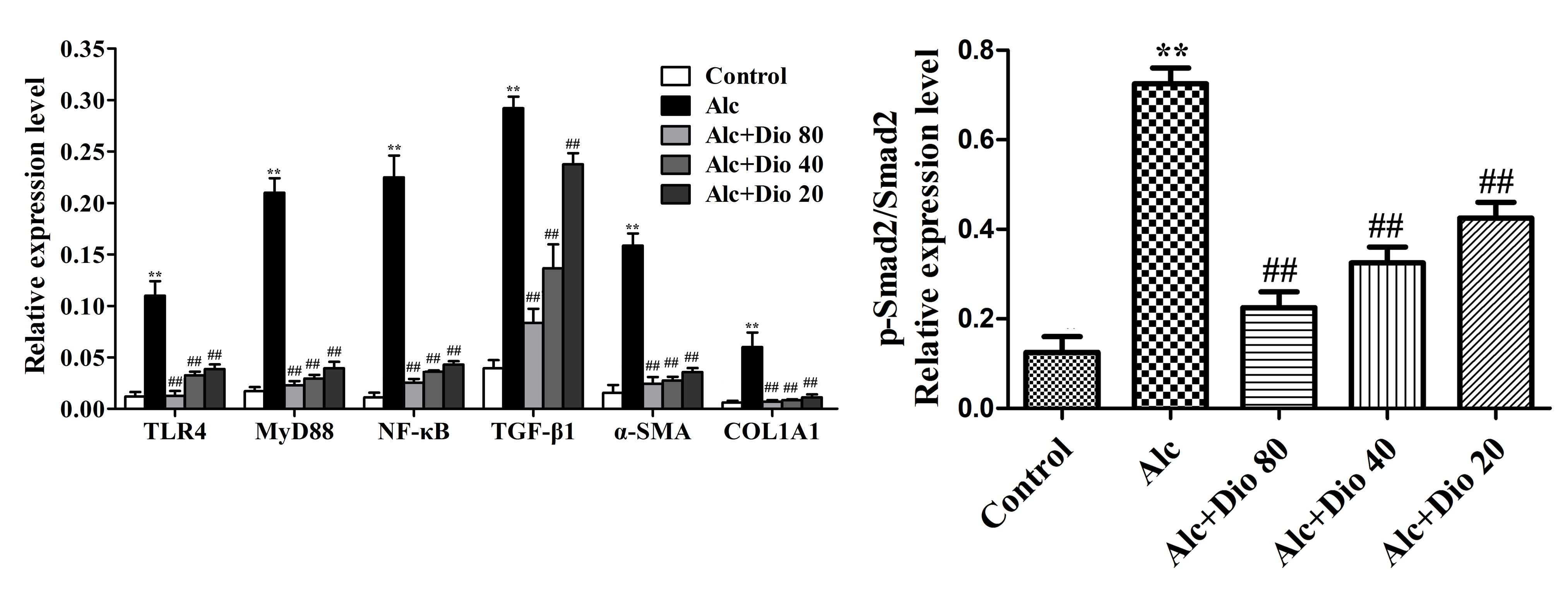


Supplemental Figure 2. Effects of dioscin on the levels of TLR4, MyD88, NF-κB, TGF-β1, p-Smad2/Smad2, α-SMA and COL1A1 measured by Western blotting in C57BL/6J mice. The values are expressed as the mean ± SD (n = 3). *p < 0.05 and **p < 0.01 vs. control group, ^#^p < 0.05 and ^##^p < 0.01 vs. model group.


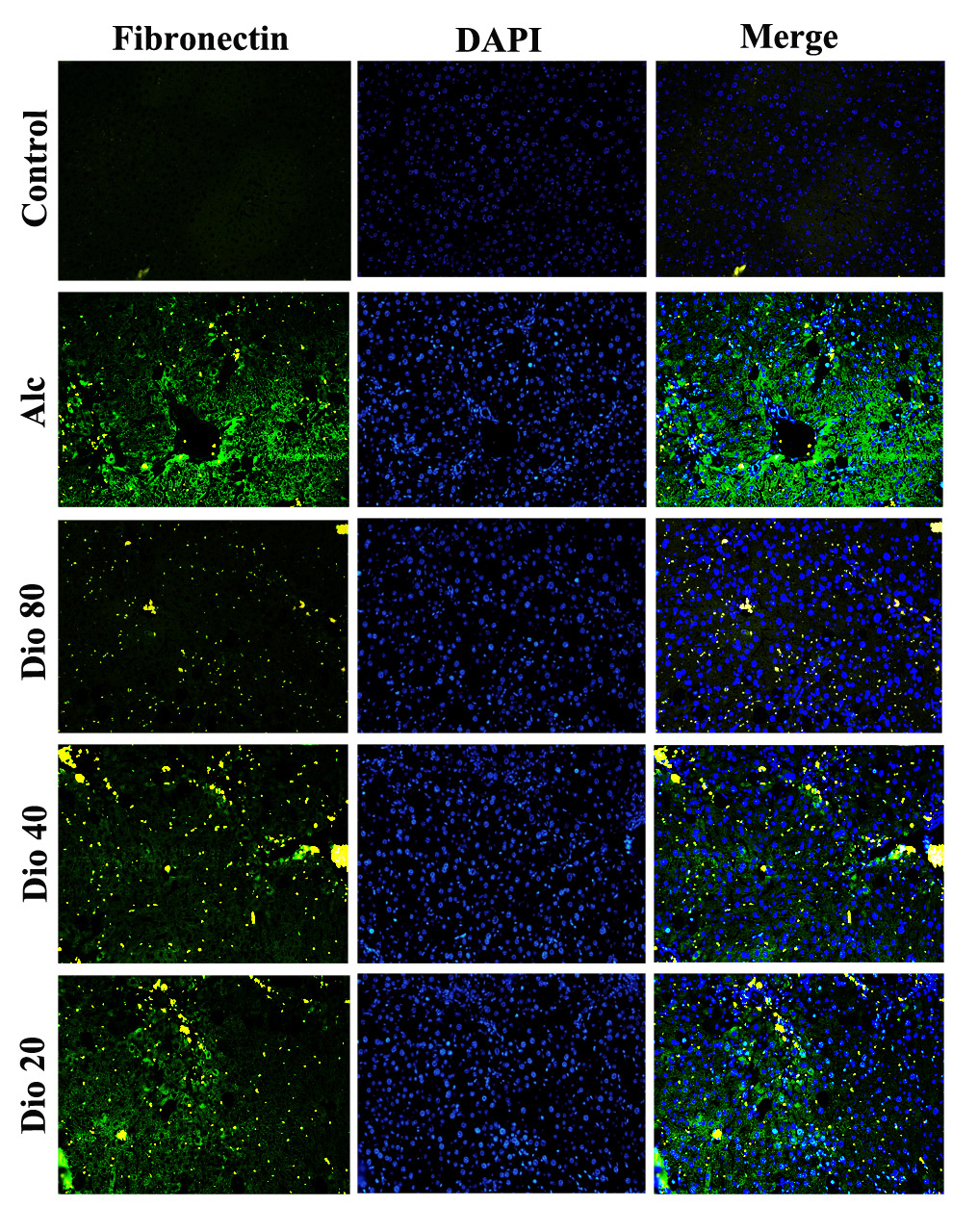


Supplemental Figure 3. Effects of dioscin on fibronectin based on immunoﬂuorescent staining (100 × original magnification).


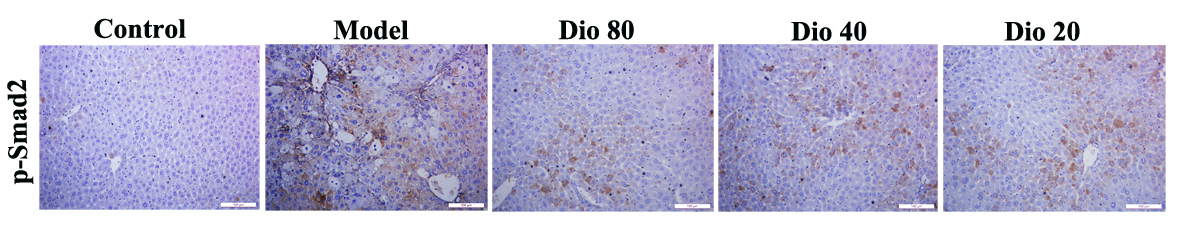


Supplemental Figure 4. Effects of dioscin on p-Smad2 based on immunohistochem- istry staining (100 × original magnification).


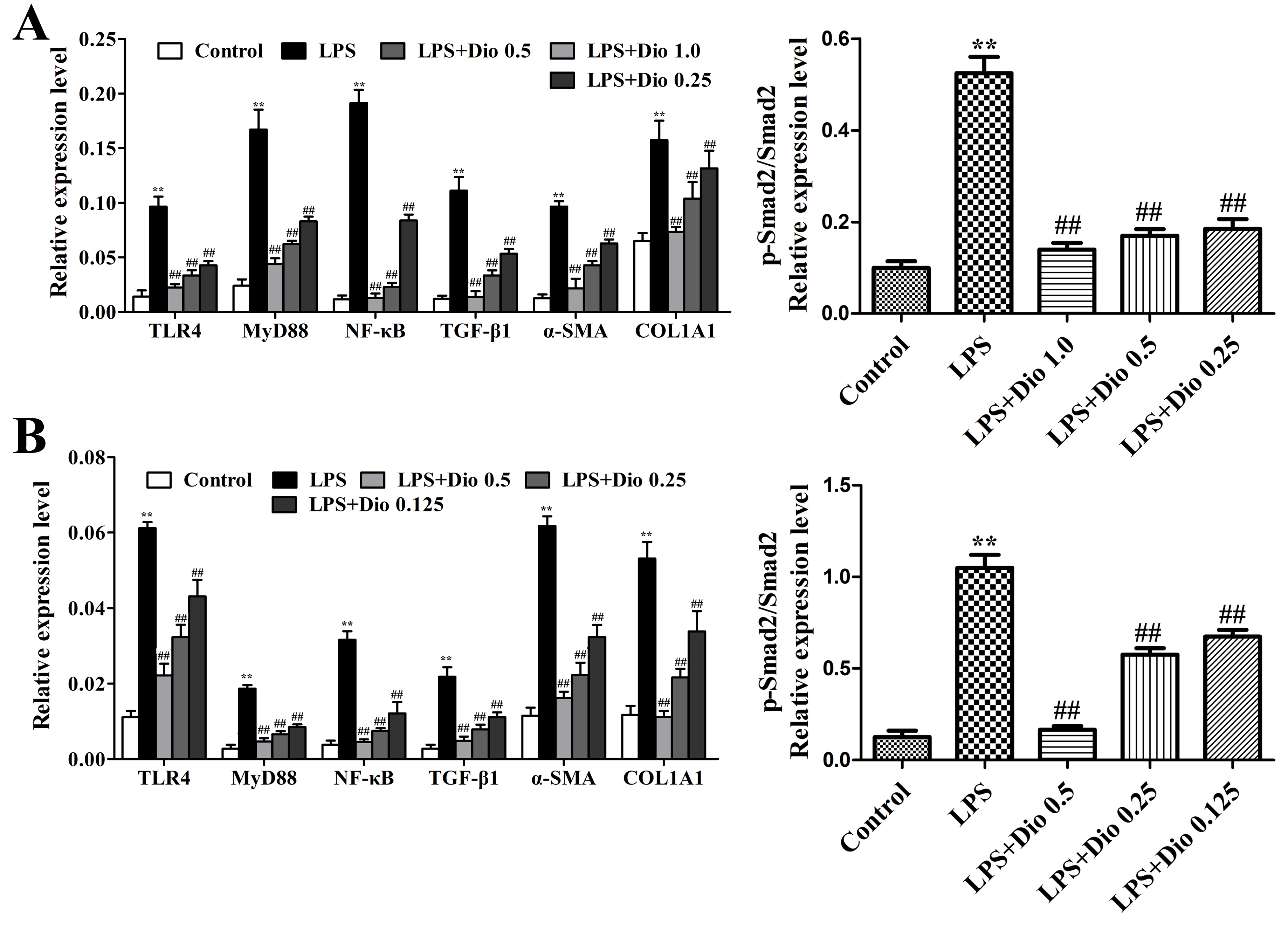


Supplemental Figure 5. (A-B) Effects of dioscin on the levels of TLR4, MyD88, NF-κB, TGF-β1, p-Smad2/Smad2, α-SMA and COL1A1 measured by Western blotting in HSC-T6 and LX2 cells. The values are expressed as the mean ± SD (n = 3). *p < 0.05 and **p < 0.01 vs. control group, ^#^p < 0.05 and ^##^p < 0.01 vs. model group.


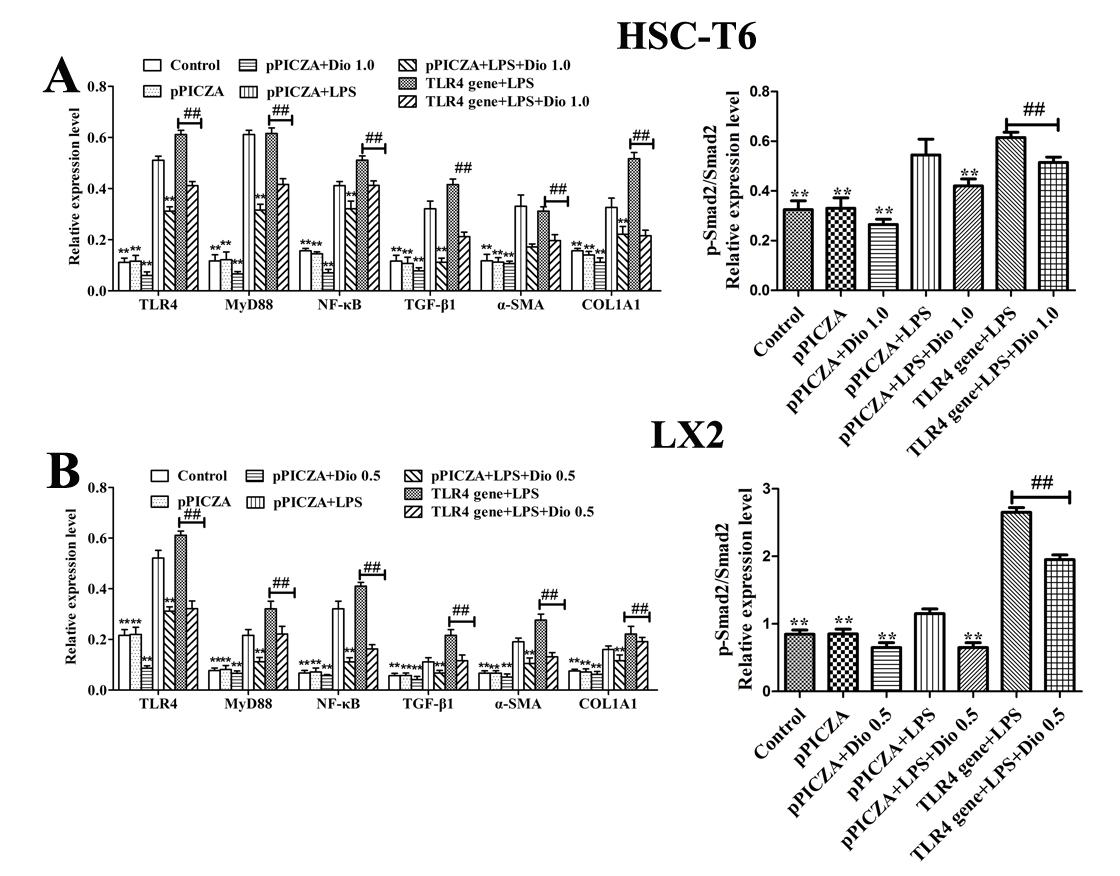


Supplemental Figure 6. (A-B) Effects of dioscin on the levels of TLR4, MyD88, NF-κB, TGF-β1, p-Smad2/Smad2, α-SMA, and COL1A1 with or without the TLR4 gene or pPICZA in HSC-T6 and LX2 cells. The values are expressed as the mean ± SD (n = 3). *p < 0.05 and **p < 0.01 vs. the model group; and **^##^**p < 0.01 vs. the LPS + TLR4 gene.


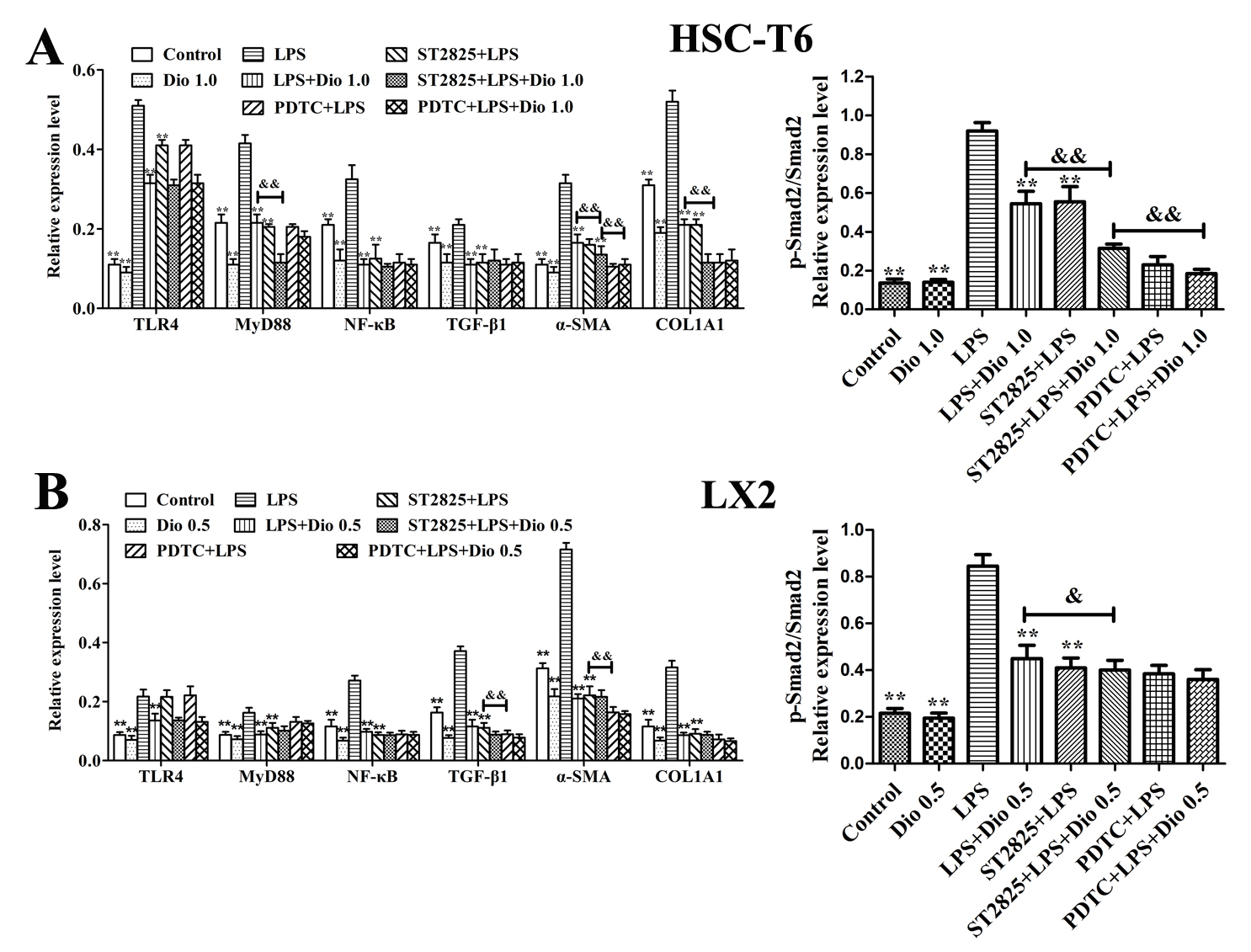


Supplemental Figure 7 (A-B) Effects of dioscin on the levels of TLR4, MyD88, NF-κB, TGF-β1, p-Smad2/ Smad2, α-SMA and COL1A1 with or without ST2825 and PDTC in HSC-T6 and LX2 cells. The values are expressed as the mean ± SD (n = 3). *p < 0.05 and**p < 0.01 vs. the model group; **^&^**p < 0.05 and **^&&^**p < 0.01 vs. LPS + Dio + ST2825.


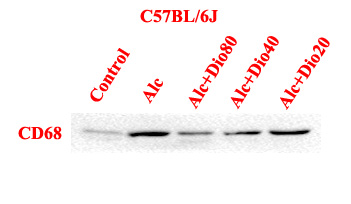


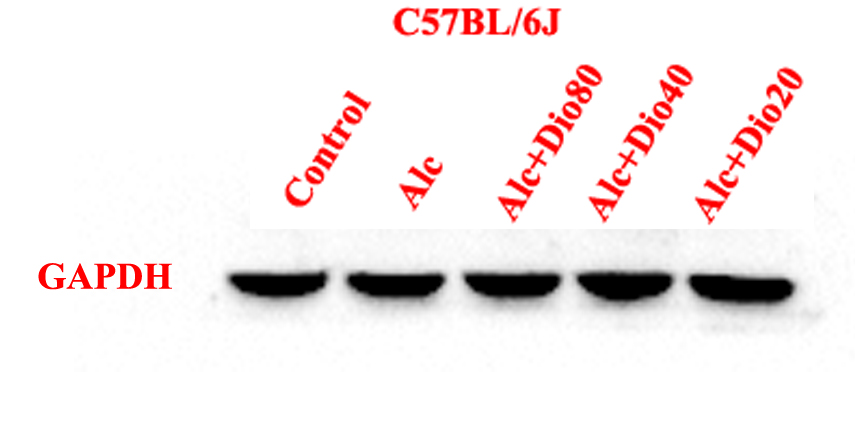


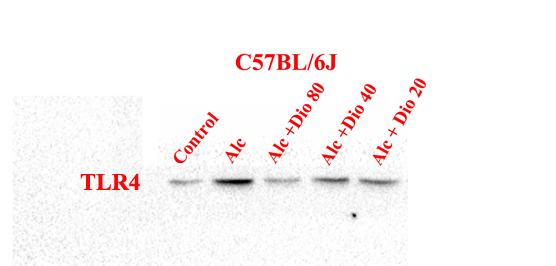


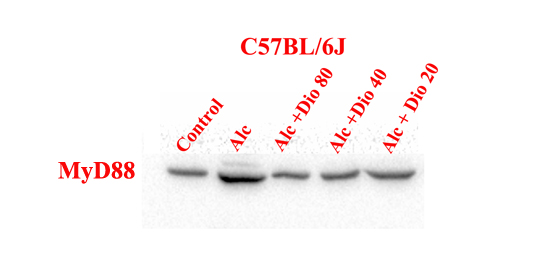

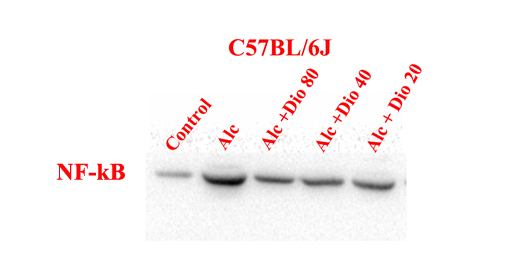


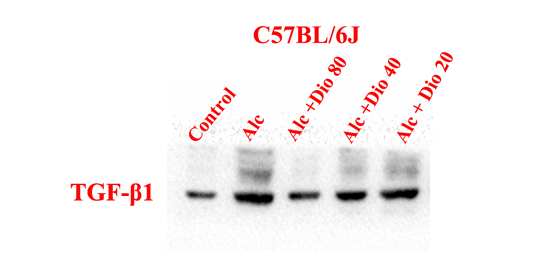


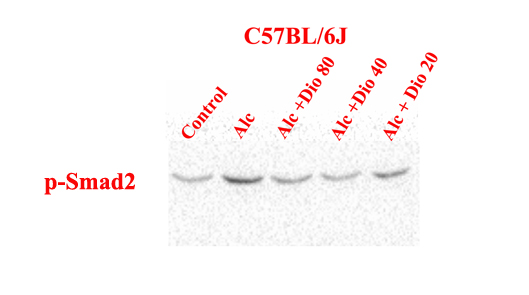


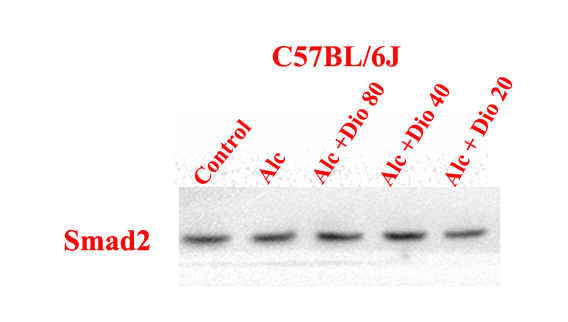


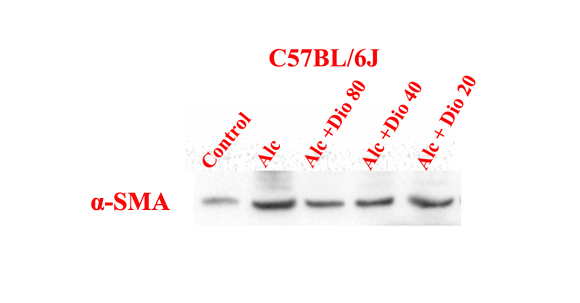


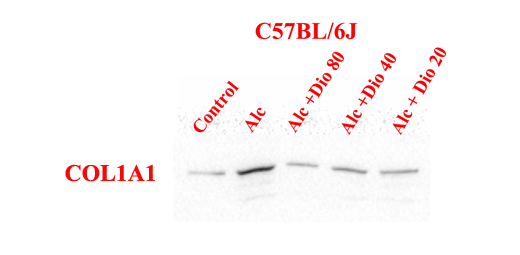


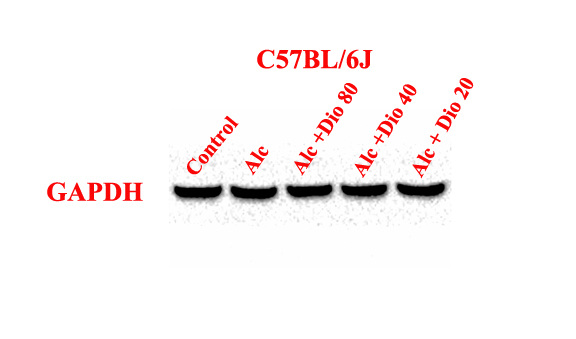


Supplemental Figure 8. Effects of dioscin on the expression of proteins related to inflammation, including TLR4, MyD88, NF-kB, TGF-β1, p-Smad2, Smad2, α-SMA, COL1A1 in mice.


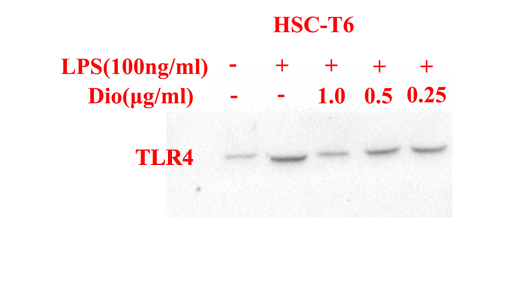


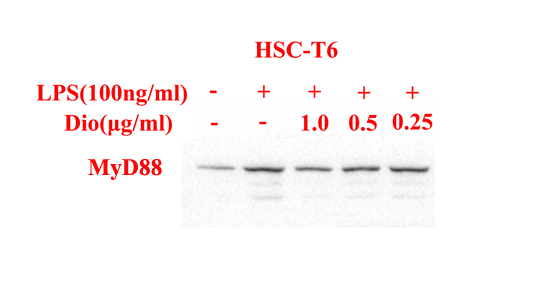


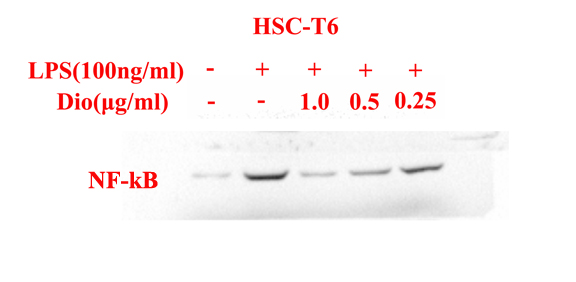


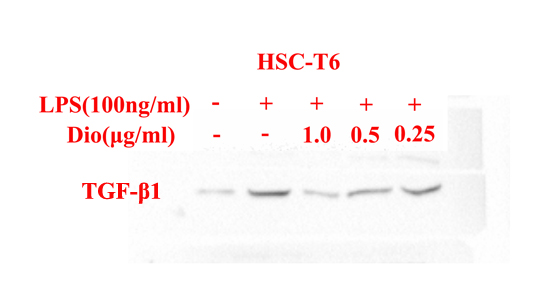


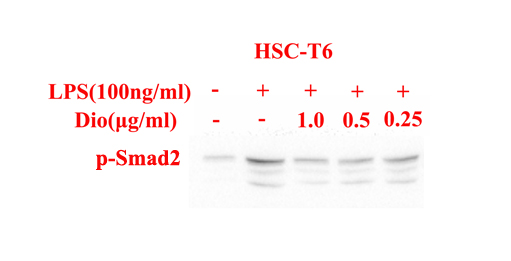


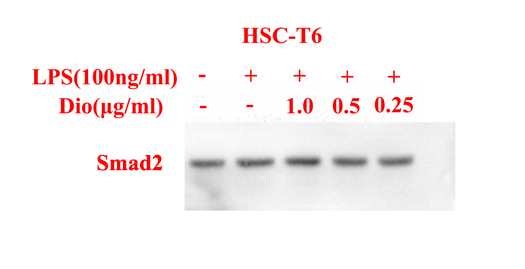


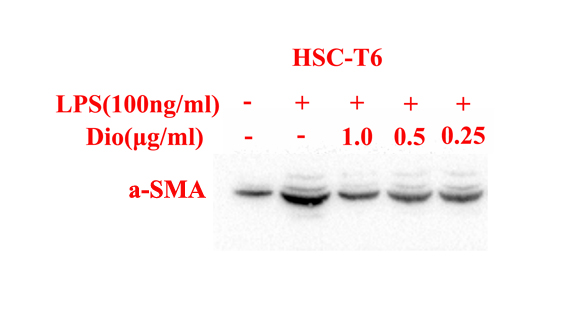


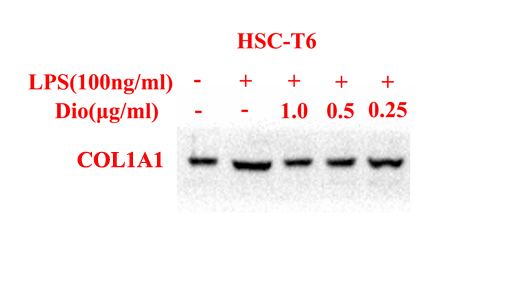


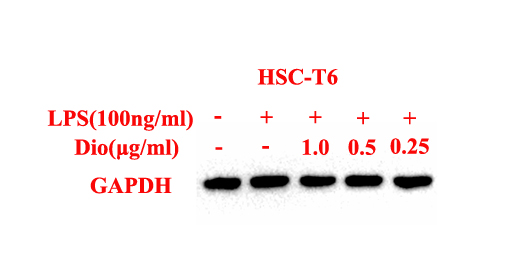


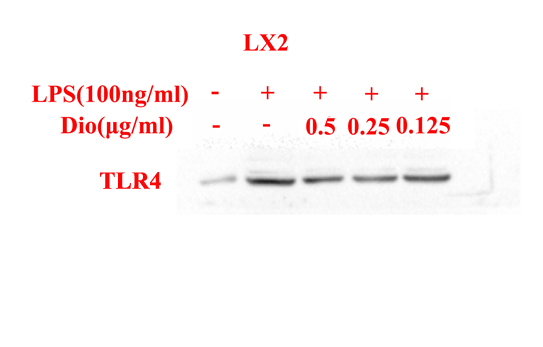


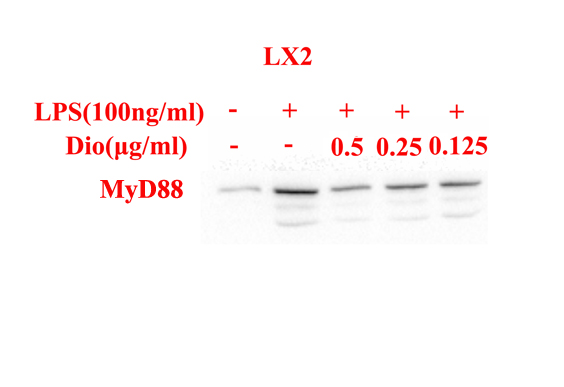


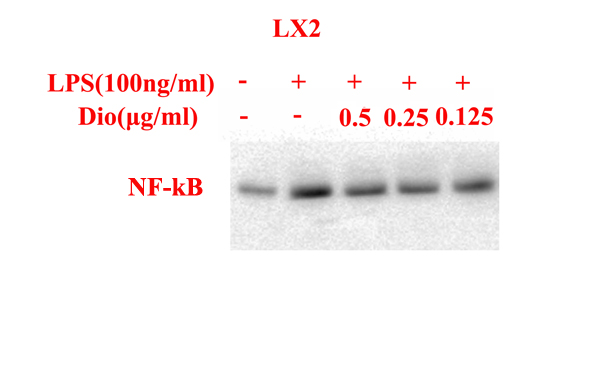


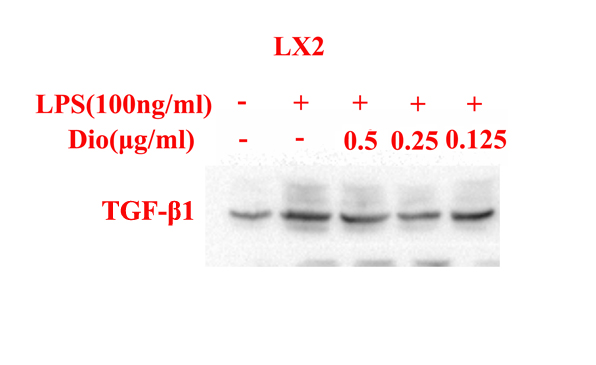


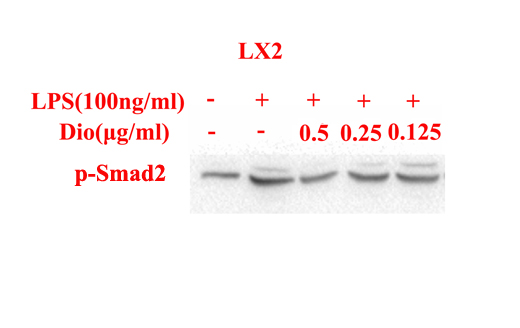


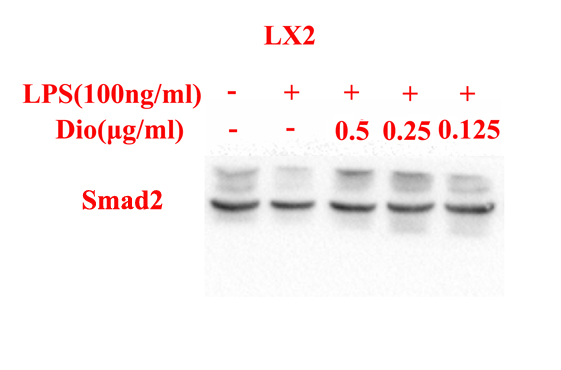


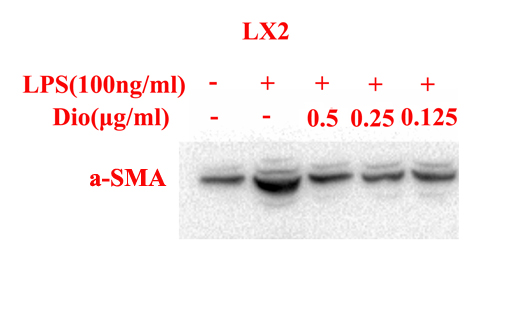


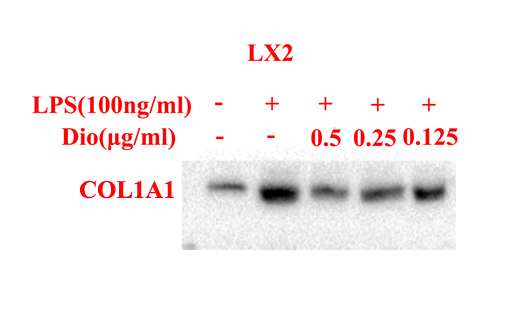


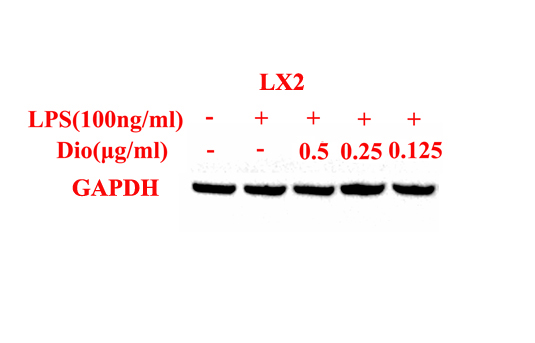


Supplemental Figure 9. Effects of dioscin on the expression of proteins related to inflammation, including TLR4, MyD88, NF-kB, TGF-β1, p-Smad2, Smad2, α-SMA, COL1A1 in HSC-T6 and LX2 cells.


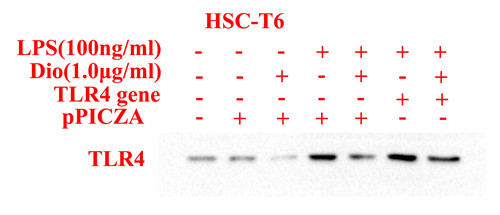


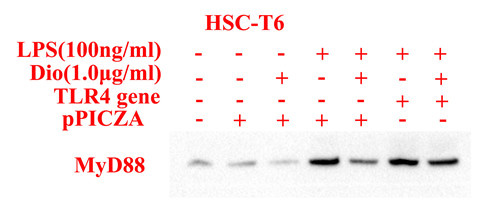

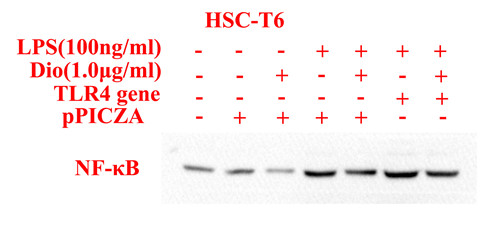

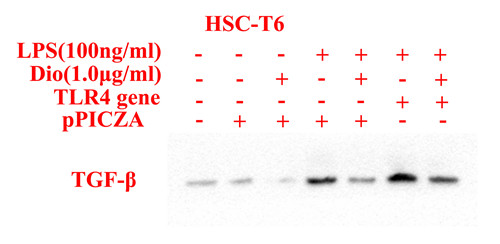


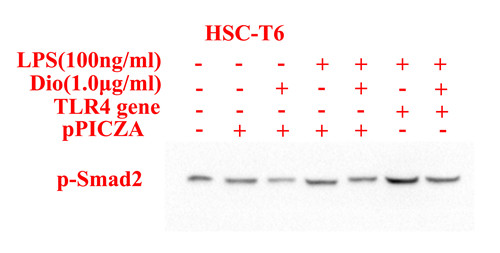


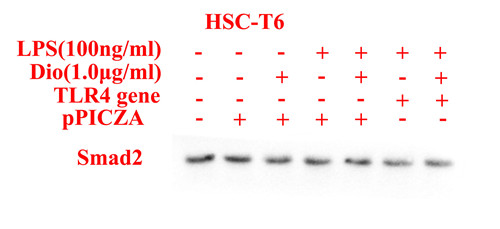

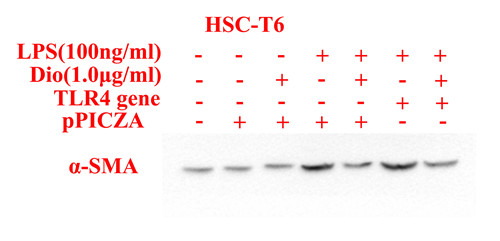


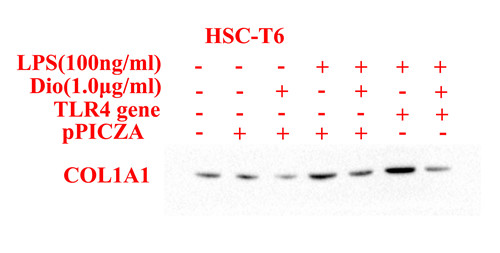


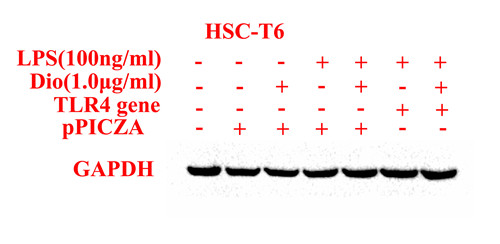

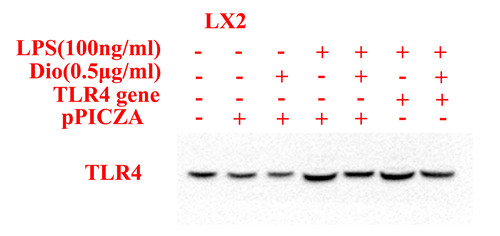

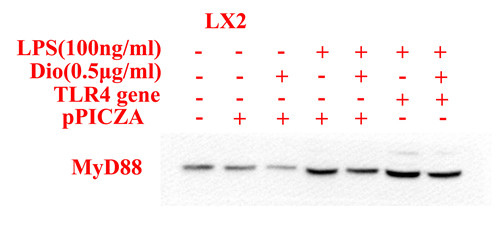

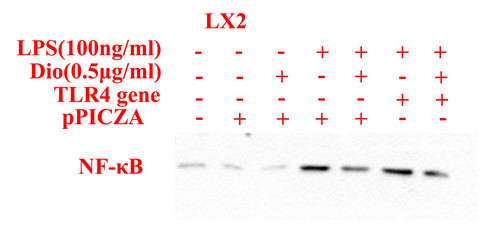

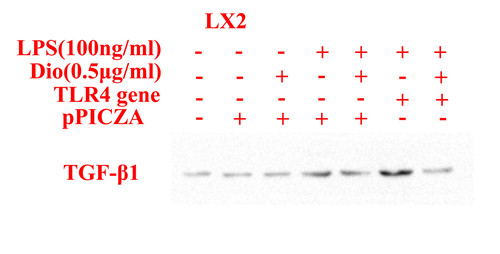

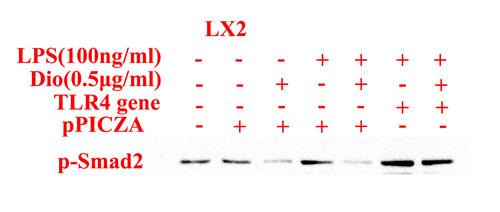

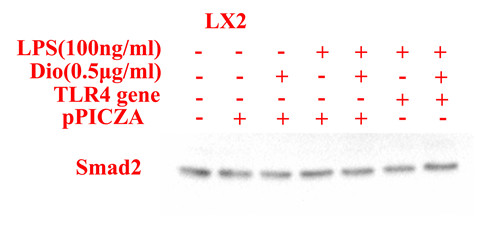

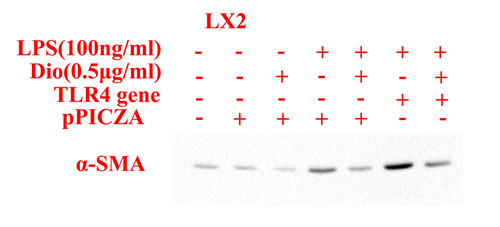

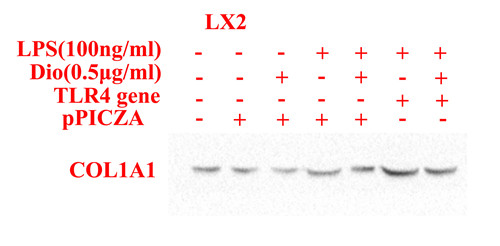

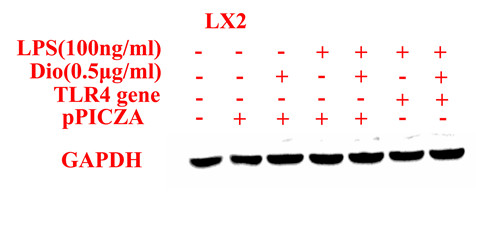


Supplemental Figure 10. Effects of dioscin on the expression of TLR4, MyD88, NF-κB, TGF-β1, p-Smad2/ Smad2, α-SMA, COL1A1 with or without TLR4 gene or pPICZA in HSC-T6 and LX2 cells.


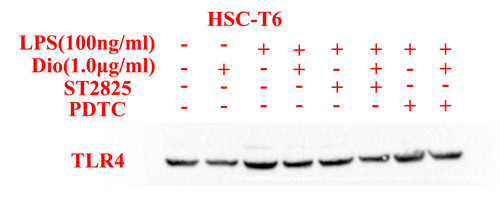

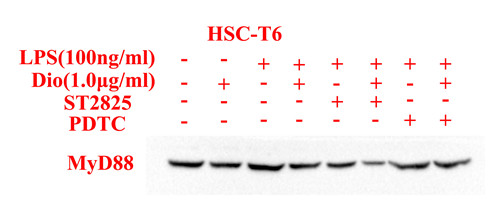

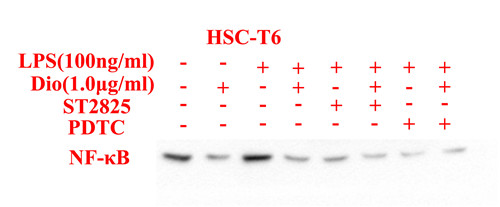

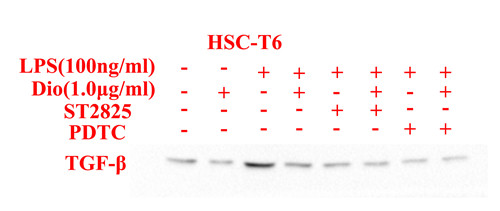

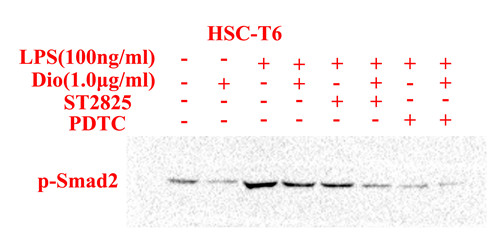

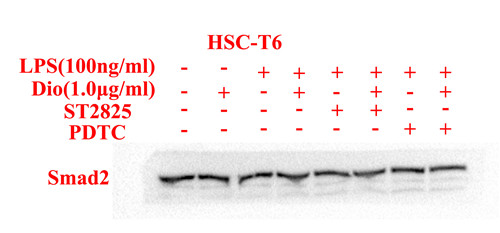

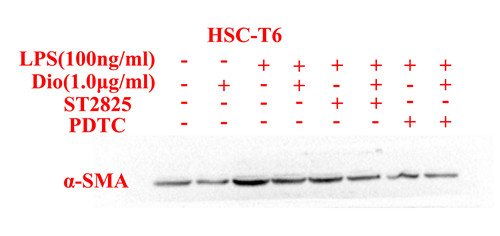

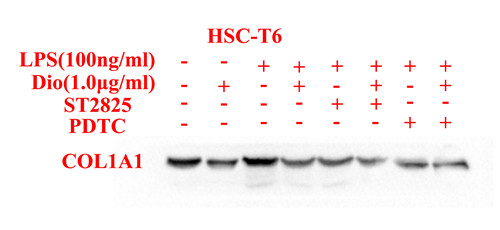

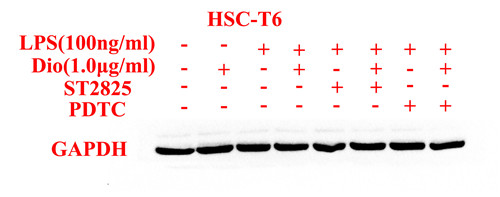

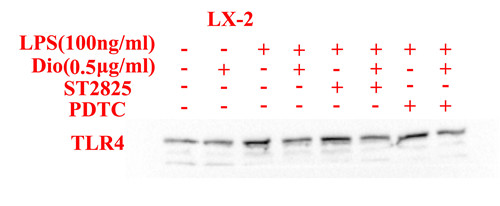

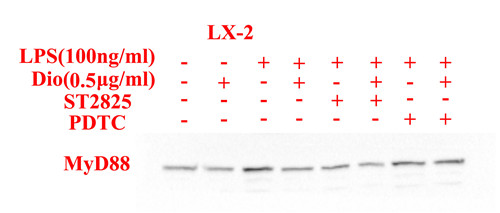

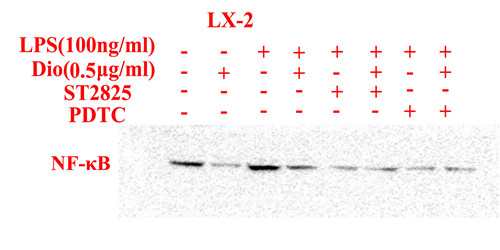

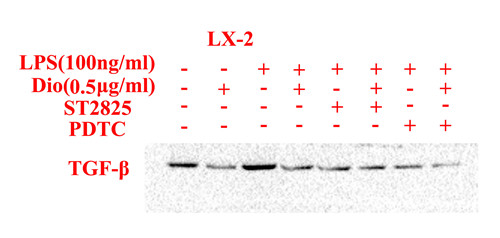

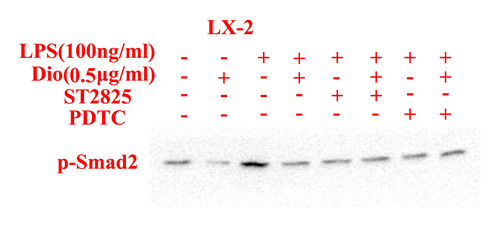

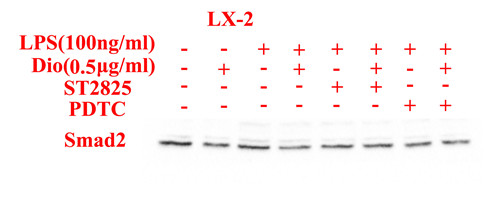

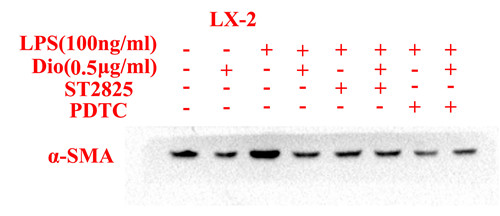

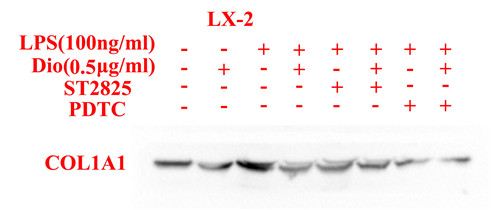

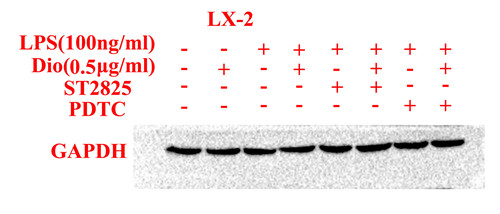


Supplemental Figure 11. Effects of dioscin on the expression of TLR4, MyD88, NF-κB, TGF-β1, p-Smad2/ Smad2, α-SMA, COL1A1 with or without ST2825, PDTC in HSC -T6 and LX2 cells.
